# Supplementary material for: Synthesis and Antiproliferative Evaluation of Novel Longifolene-Derived Tetralone Derivatives Bearing 1,2,4-Triazole Moiety
Source: Molecules. 2020 Feb 22;25(4):986. doi: 10.3390/molecules25040986 (PMC7070458; doi:10.3390/molecules25040986)
Supplement: Supplementary file 1 [file molecules-25-00986-s001.pdf]

Synthesis and Antiproliferative Evaluation of Novel Longifolene-Derived Tetralone Derivatives Bearing 1,2,4-Triazole Moiety

Xia-Ping Zhu [1], Ggu-Shan Lin [1,*], Wen-Gui Duan [1,*], Qing-Min Li[1], Fang-Yao Li [2] and Shun-Zhong Lu [3]

Contents

Figure S1. FT-IR spectrum of compound **2**....4

Figure S2. ^{1}H-NMR spectrum of compound **2** in $CDCl_3$....4

Figure S3. ^{13}C-NMR spectrum of compound **2** in $CDCl_3$....4

Figure S4. HMBC spectrum of compound **2** in $CDCl_3$....5

Figure S5. ESI-MS spectrum of compound **2**....5

Figure S6. FT-IR spectrum of compound **3**....5

Figure S7. ^{1}H-NMR spectrum of compound **3** in $CDCl_3$....6

Figure S8. ^{13}C-NMR spectrum of compound **3** in $CDCl_3$....6

Figure S9. ESI-MS spectrum of compound **3**....6

Figure S10. FT-IR spectrum of compound **4**....7

Figure S11. ^{1}H-NMR spectrum of compound **4** in $CDCl_3$....7

Figure S12. ^{13}C-NMR spectrum of compound **4** in $CDCl_3$....7

Figure S13. ESI-MS spectrum of compound **4**....8

Figure S14. FT-IR spectrum of compound **5**....8

Figure S15. ^{1}H-NMR spectrum of compound **5** in DMSO-d_6....8

Figure S16. ^{13}C-NMR spectrum of compound **5** in DMSO-d_6....9

Figure S17. ESI-MS spectrum of compound **5**....9

Figure S18. FT-IR spectrum of the target compound **6a**....9

Figure S19. ^{1}H-NMR spectrum of the target compound **6a** in $CDCl_3$....10

Figure S20. ^{13}C-NMR spectrum of the target compound **6a** in $CDCl_3$....10

Figure S21. NOESY spectrum of compound **6a** in $CDCl_3$. 10

Figure S22. ESI-MS spectrum of the target compound **6a**....11

Figure S23. FT-IR spectrum of the target compound **6b**....11

Figure S24. ^{1}H-NMR spectrum of the target compound **6b** in DMSO-d_6....11

Figure S25. ^{13}C-NMR spectrum of the target compound **6b** in DMSO-d_6....12

Figure S26. ESI-MS spectrum of the target compound **6b**....12

Figure S27. FT-IR spectru m of the target compound **6c**....12

Figure S28. ^{1}H-NMR spectrum of the target compound **6c** in DMSO-d_6....13

Figure S29. ^{13}C-NMR spectrum of the target compound **6c** in DMSO-d_6 13
Figure S30. ESI-MS spectrum of the target compound **6c** 13
Figure S31. FT-IR spectrum of the target compound **6d** 14
Figure S32. ^{1}H-NMR spectrum of the target compound **6d** in DMSO-d_6 14
Figure S33. ^{13}C-NMR spectrum of the target compound **6d** in DMSO-d_6 14
Figure S34. ESI-MS spectrum of the target compound **6d** 15
Figure S35. FT-IR spectrum of the target compound **6e** 15
Figure S36. ^{1}H-NMR spectrum of the target compound **6e** in $CDCl_3$ 15
Figure S37. ^{13}C-NMR spectrum of the target compound **6e** in $CDCl_3$ 16
Figure S38. ESI-MS spectrum of the target compound **6e** 16
Figure S39. FT-IR spectrum of the target compound **6f** 16
Figure S40. ^{1}H-NMR spectrum of the target compound **6f** in DMSO-d_6 17
Figure S41. ^{13}C-NMR spectrum of the target compound **6f** in DMSO-d_6 17
Figure S42. ESI-MS spectrum of the target compound **6f** 17
Figure S43. FT-IR spectrum of the target compound **6g** 18
Figure S44. ^{1}H-NMR spectrum of the target compound **6g** in $CDCl_3$ 18
Figure S45. ^{13}C-NMR spectrum of the target compound **6g** in $CDCl_3$ 18
Figure S46. ESI-MS spectrum of the target compound **6g** 19
Figure S47. FT-IR spectrum of the target compound **6h** 19
Figure S48. ^{1}H-NMR spectrum of the target compound **6h** in DMSO-d_6 19
Figure S49. ^{13}C-NMR spectrum of the target compound **6h** in DMSO-d_6 20
Figure S50. ESI-MS spectrum of the target compound **6h** 20
Figure S51. FT-IR spectrum of the target compound **6i** 20
Figure S52. ^{1}H-NMR spectrum of the target compound **6i** in DMSO-d_6 21
Figure S53. ^{13}C-NMR spectrum of the target compound **6i** in DMSO-d_6 21
Figure S54. ESI-MS spectrum of the target compound **6i** 21
Figure S55. FT-IR spectrum of the target compound **6j** 22
Figure S56. ^{1}H-NMR spectrum of the target compound **6j** in DMSO-d_6 22
Figure S57. ^{13}C-NMR spectrum of the target compound **6j** in DMSO-d_6 22
Figure S58. ESI-MS spectrum of the target compound **6j** 23
Figure S59. FT-IR spectrum of the target compound **6k** 23
Figure S60. ^{1}H-NMR spectrum of the target compound **6k** in DMSO-d_6 23
Figure S61. ^{13}C-NMR spectrum of the target compound **6k** in DMSO-d_6 24
Figure S62. ESI-MS spectrum of the target compound **6k** 24
Figure S63. FT-IR spectrum of the target compound **6l** 24

Figure S64. ^{1}H-NMR spectrum of the target compound **6l** in $CDCl_3$ 25

Figure S65. ^{13}C-NMR spectrum of the target compound **6l** in $CDCl_3$ 25

Figure S66. ESI-MS spectrum of the target compound **6l** 25

Figure S67. FT-IR spectrum of the target compound **6m** 26

Figure S68. ^{1}H-NMR spectrum of the target compound **6m** in DMSO-d_6 26

Figure S69. ^{13}C-NMR spectrum of the target compound **6m** in DMSO-d_6 26

Figure S70. ESI-MS spectrum of the target compound **6m** 27

Figure S71. FT-IR spectrum of the target compound **6n** 27

Figure S72. ^{1}H-NMR spectrum of the target compound **6n** in $CDCl_3$ 27

Figure S73. ^{13}C-NMR spectrum of the target compound **6n** in $CDCl_3$ 28

Figure S74. FT-IR spectrum of the target compound **6o** 28

Figure S75. ^{1}H-NMR spectrum of the target compound **6o** in DMSO-d_6 28

Figure S76. ^{13}C-NMR spectrum of the target compound **6o** in DMSO-d_6 29

Figure S77. ESI-MS spectrum of the target compound **6o** 29

Figure S78. FT-IR spectrum of the target compound **6p** 29

Figure S79. ^{1}H-NMR spectrum of the target compound **6p** in $CDCl_3$ 30

Figure S80. ^{13}C-NMR spectrum of the target compound **6p** in $CDCl_3$ 30

Figure S81. ESI-MS spectrum of the target compound **6p** 30

Figure S82. FT-IR spectrum of the target compound **6q** 31

Figure S83. ^{1}H-NMR spectrum of the target compound **6q** in $CDCl_3$ 31

Figure S84. ^{13}C-NMR spectrum of the target compound **6q** in $CDCl_3$ 32

Figure S85. ESI-MS spectrum of the target compound **6q** 32

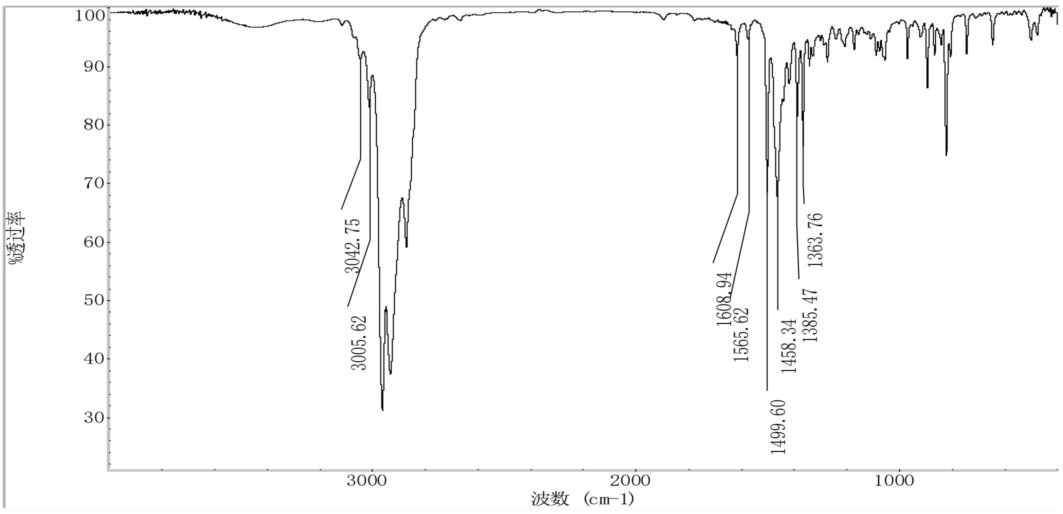

Figure S1. FT-IR spectrum of compound **2**

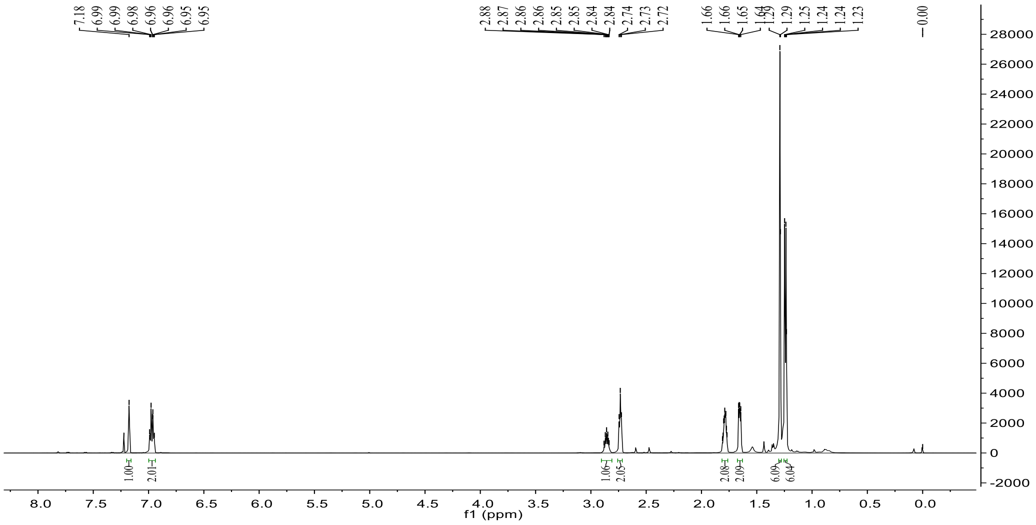

Figure S2. ^{1}H-NMR spectrum of compound **2** in $CDCl_3$

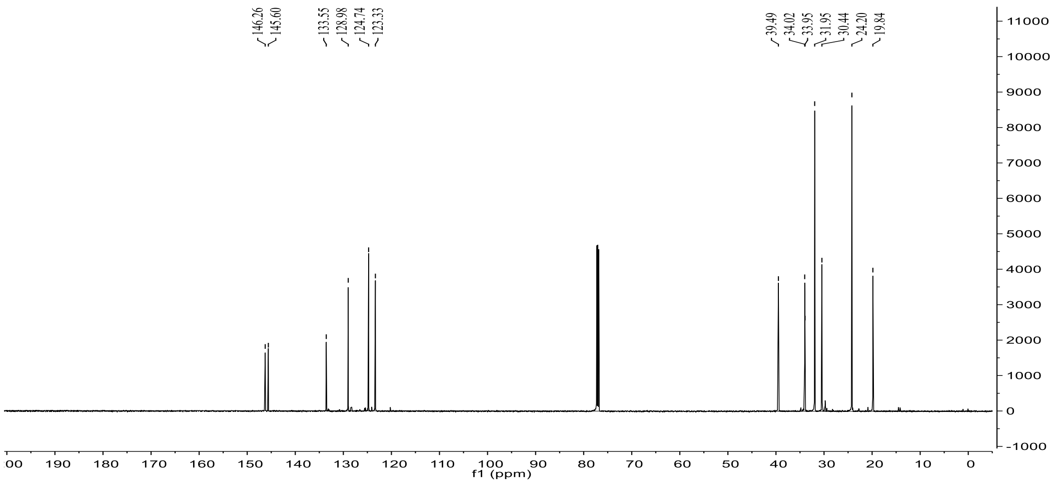

Figure S3. ^{13}C-NMR spectrum of compound **2** in $CDCl_3$

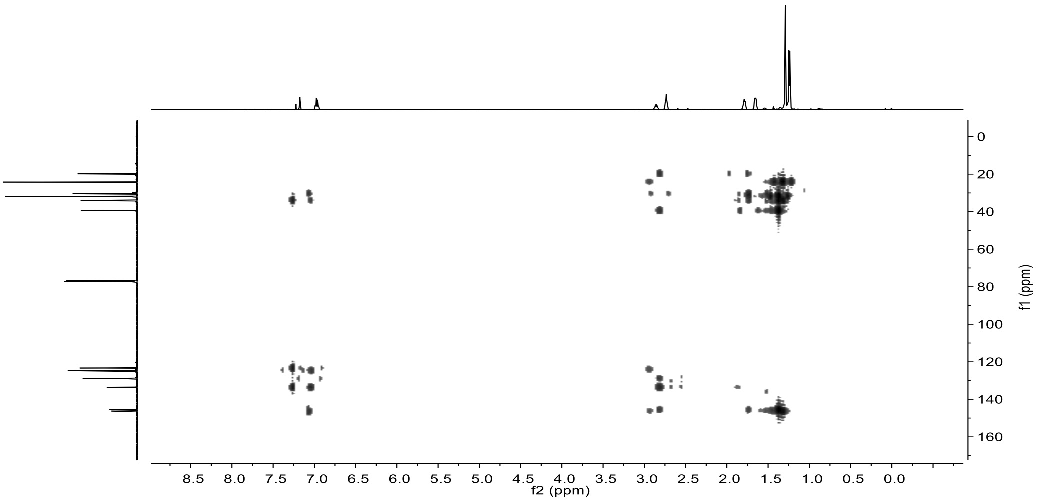

Figure S4. HMBC spectrum of compound **2** in $CDCl_3$

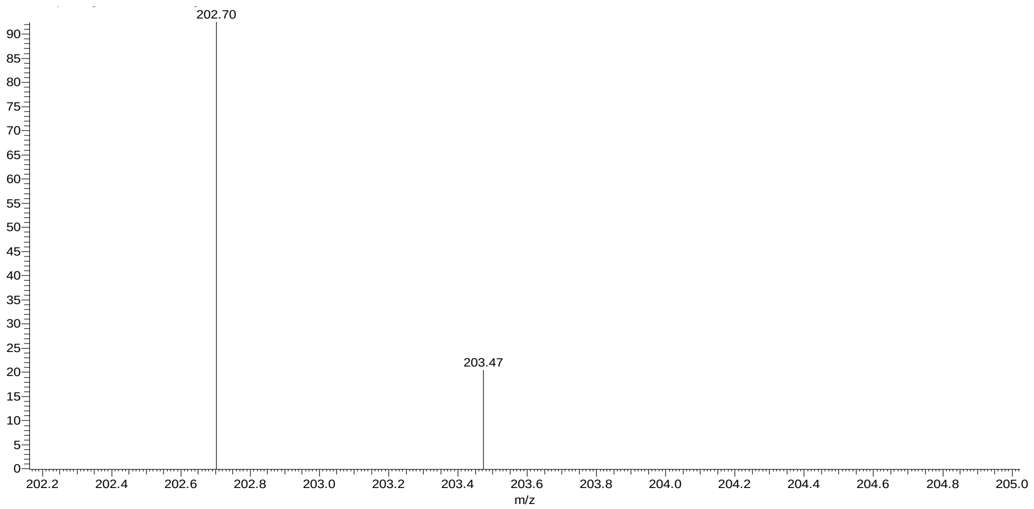

Figure S5. ESI-MS spectrum of compound **2**

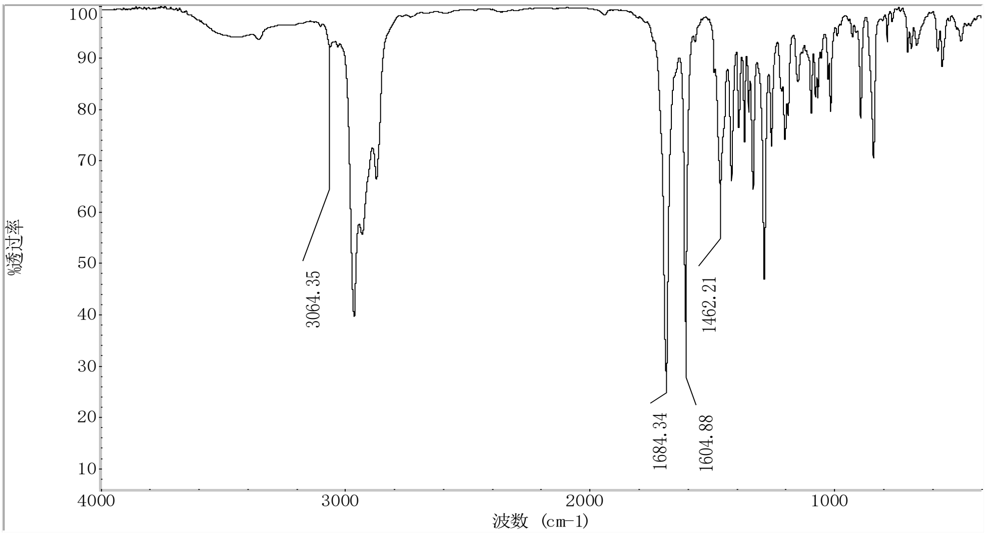

Figure S6. FT-IR spectrum of compound **3**

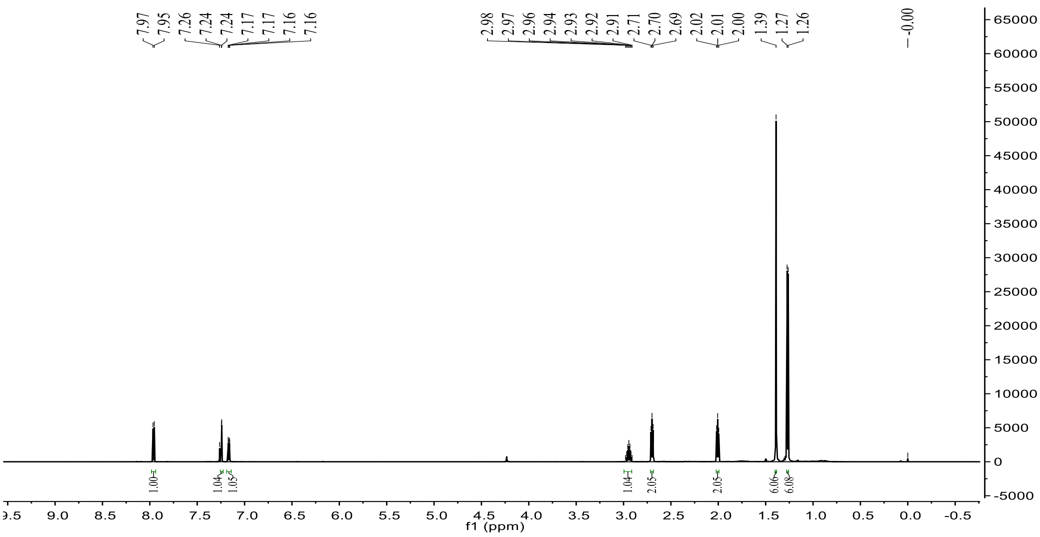

Figure S7. ^{1}H-NMR spectrum of compound **3** in $CDCl_3$

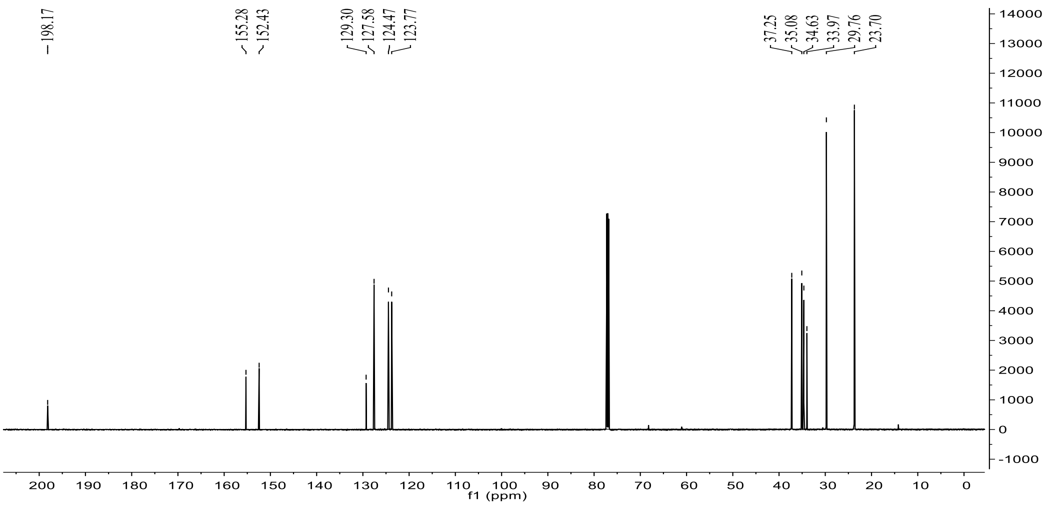

Figure S8. ^{13}C-NMR spectrum of compound **3** in $CDCl_3$

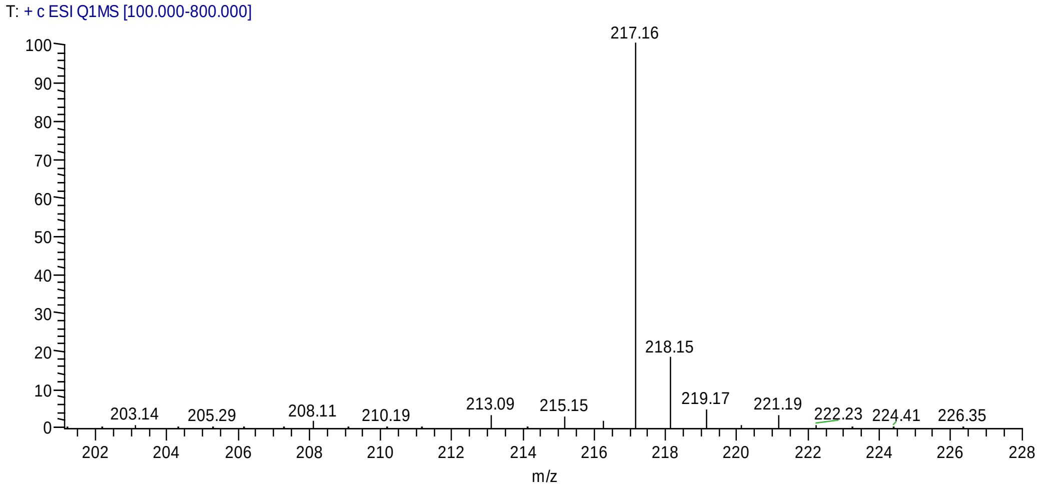

Figure S9. ESI-MS spectrum of compound **3**

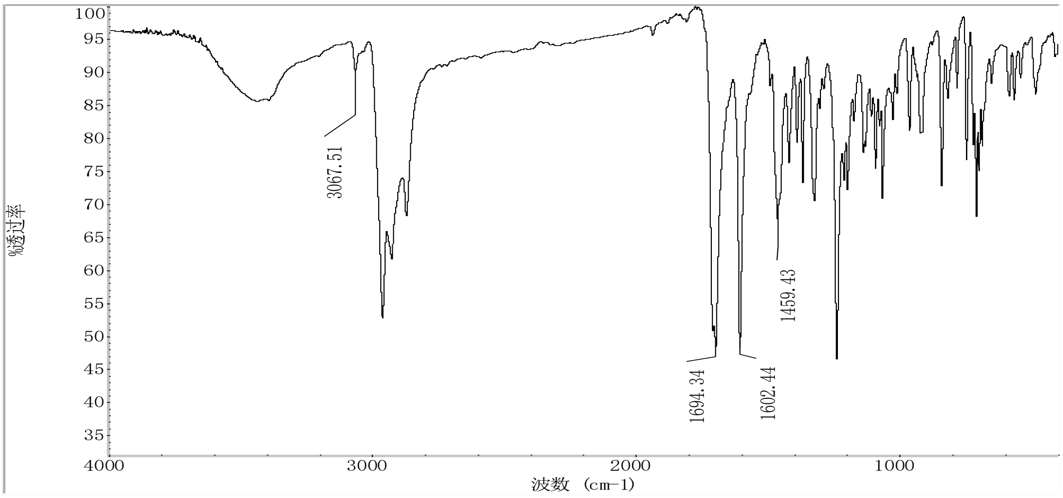

Figure S10. FT-IR spectrum of compound **4**

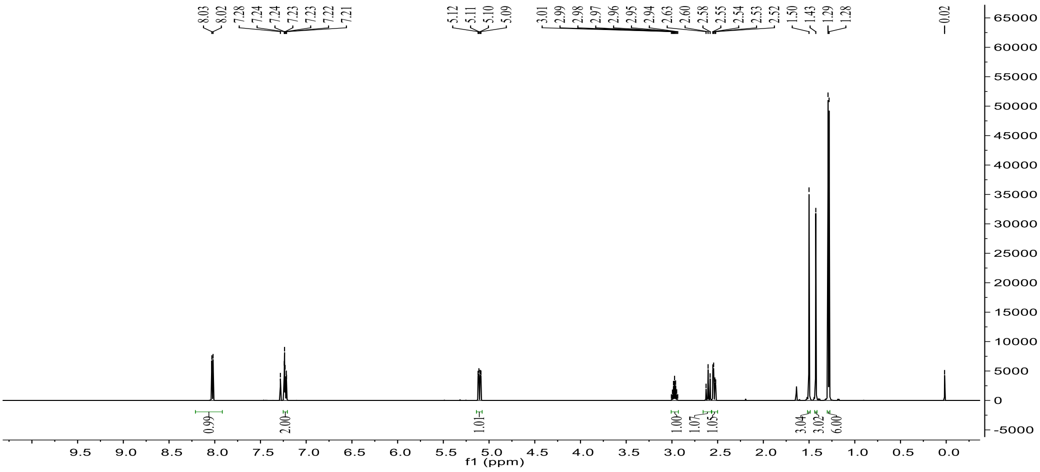

Figure S11. ^{1}H-NMR spectrum of compound **4** in $CDCl_3$

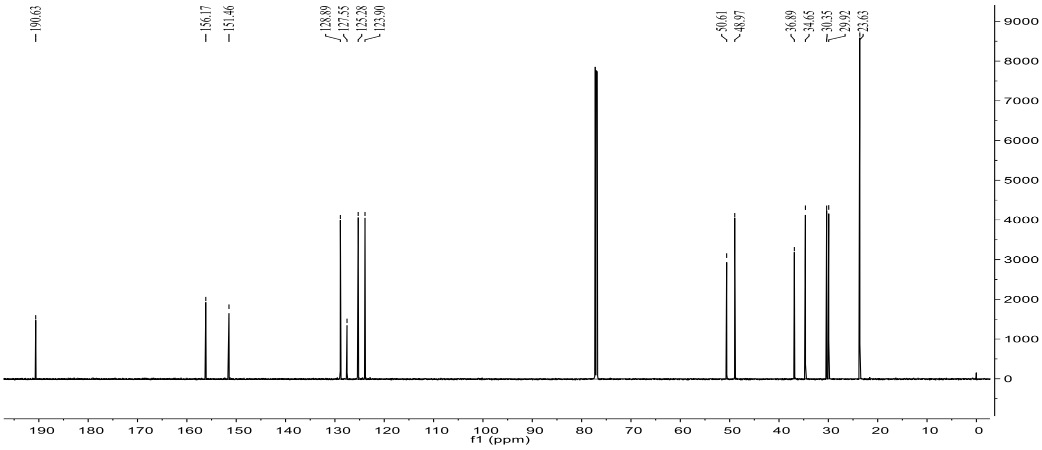

Figure S12. ^{13}C-NMR spectrum of compound **4** in $CDCl_3$

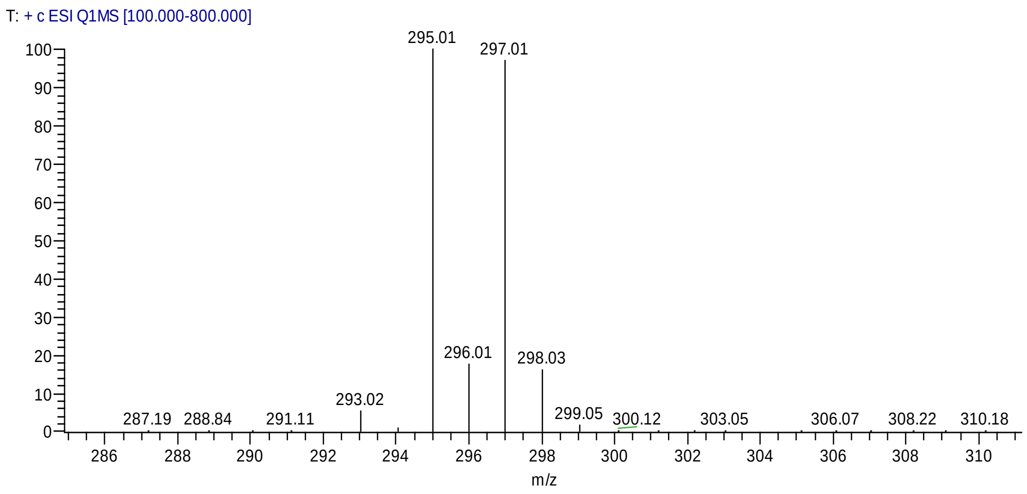

Figure S13. ESI-MS spectrum of compound **4**

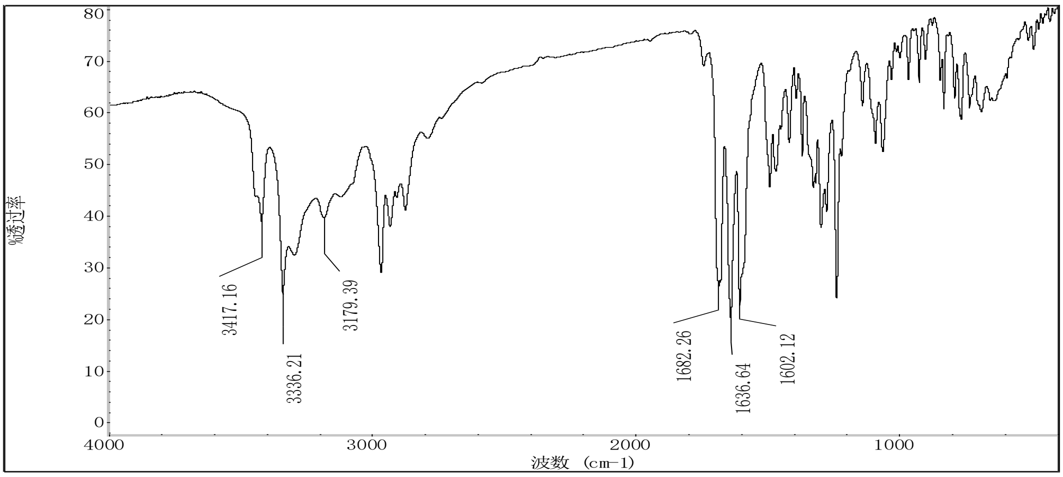

Figure S14. FT-IR spectrum of compound **5**

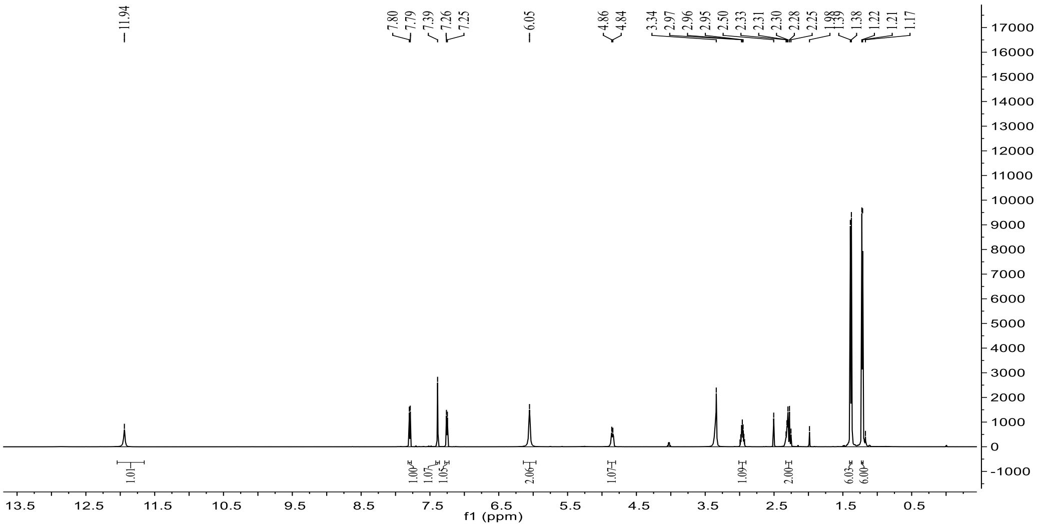

Figure S15. ^{1}H-NMR spectrum of compound **5** in DMSO-d_6

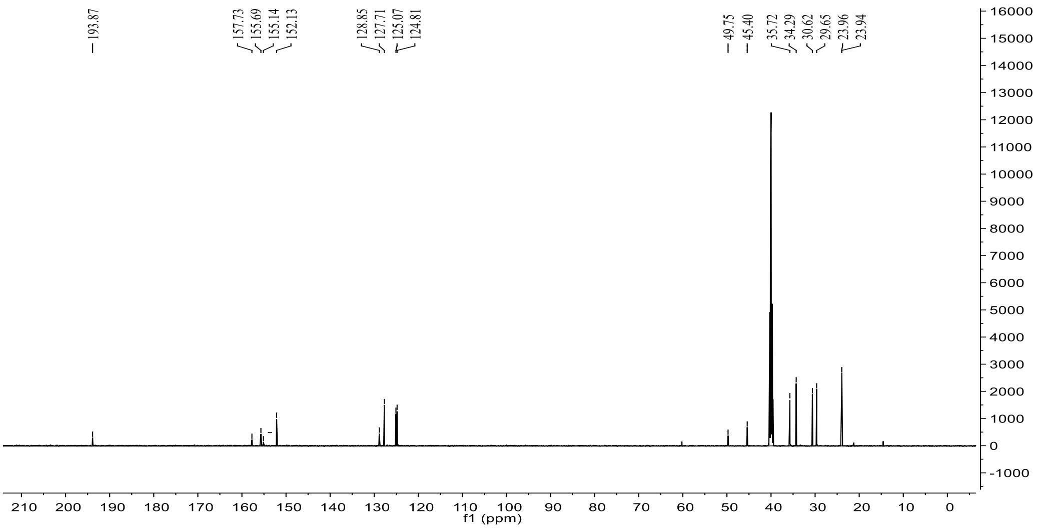

Figure S16. ^{13}C-NMR spectrum of compound **5** in DMSO-d_6

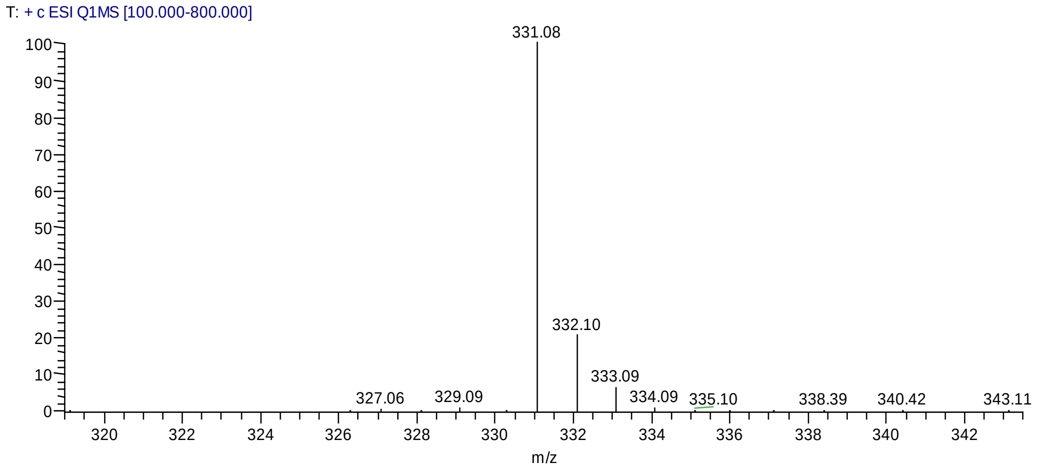

Figure S17. ESI-MS spectrum of compound **5**

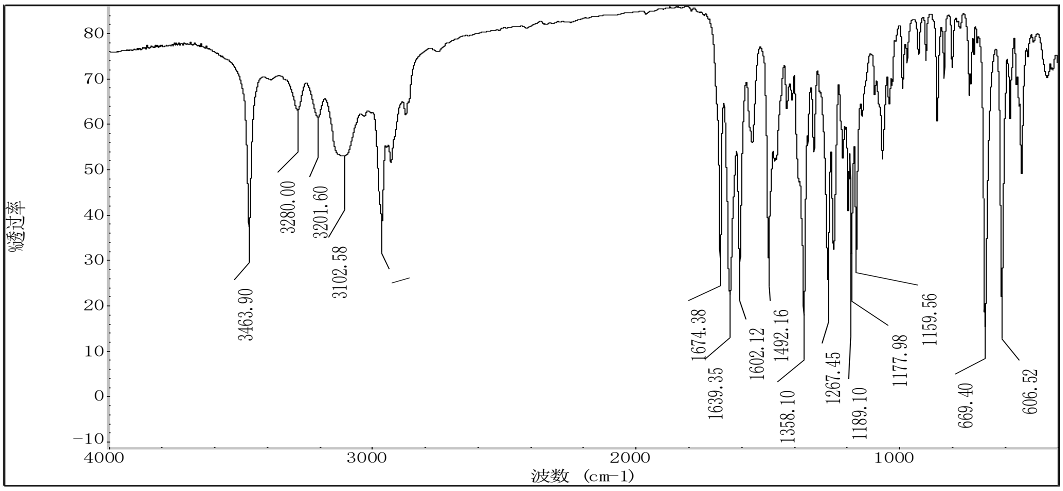

Figure S18. FT-IR spectrum of the target compound **6a**

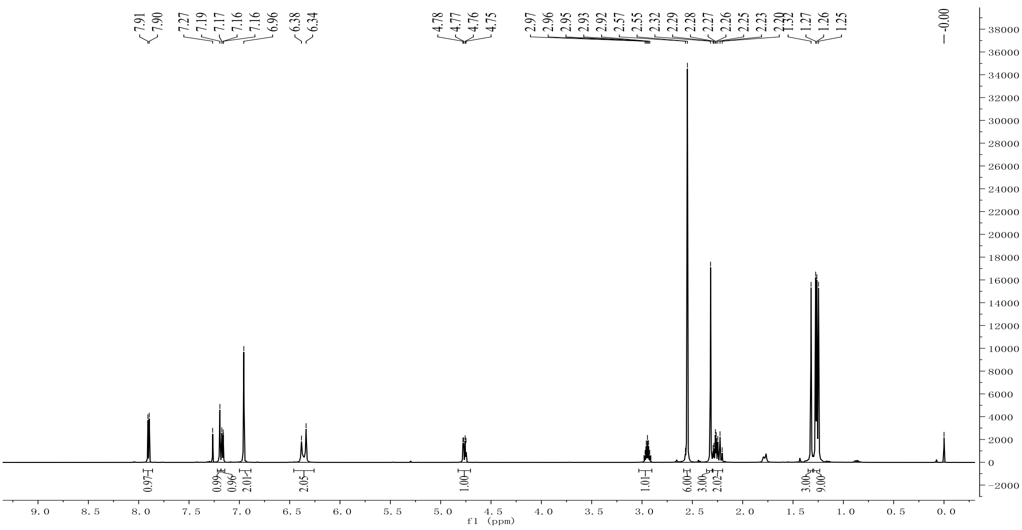

Figure S19. ^{1}H-NMR spectrum of the target compound **6a** in $CDCl_3$

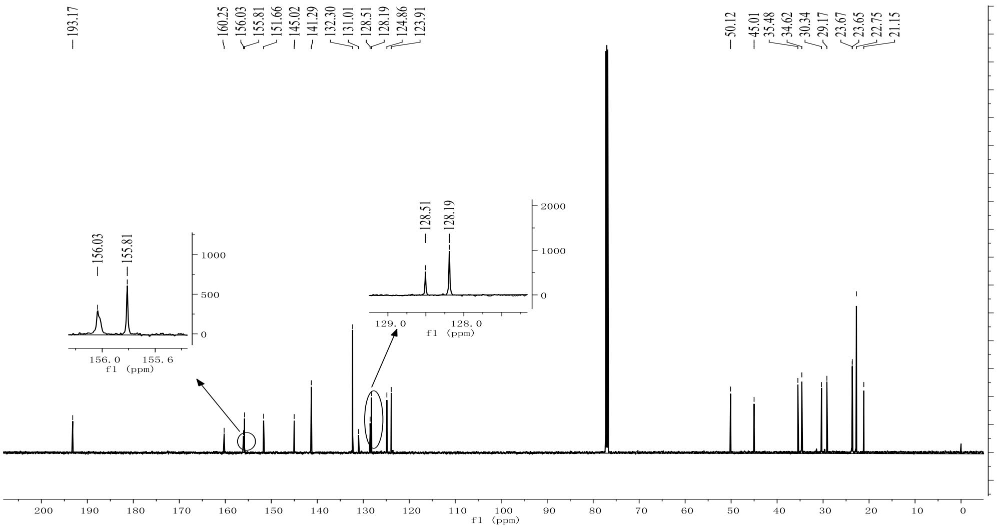

Figure S20. ^{13}C-NMR spectrum of the target compound **6a** in $CDCl_3$

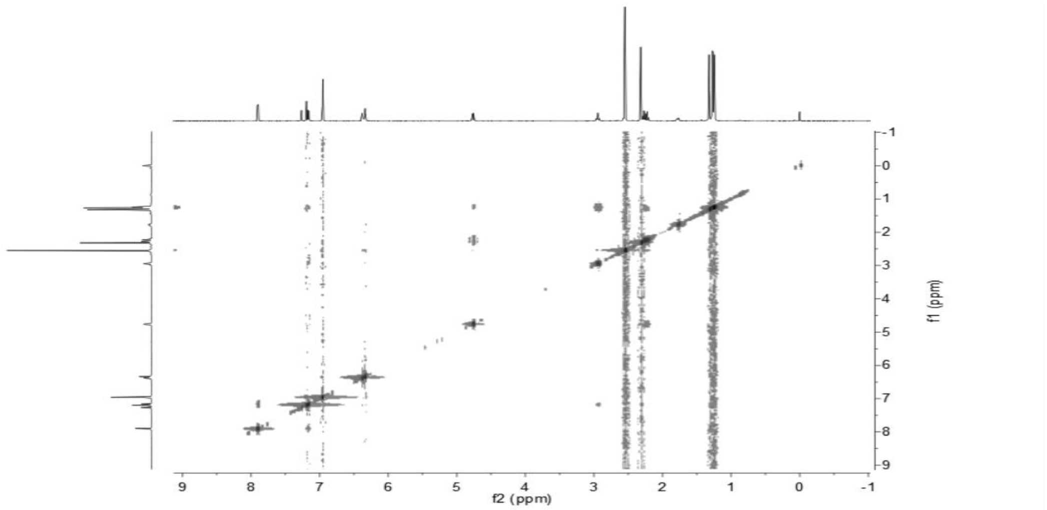

Figure S21. NOESY spectrum of compound **6a** in $CDCl_3$.

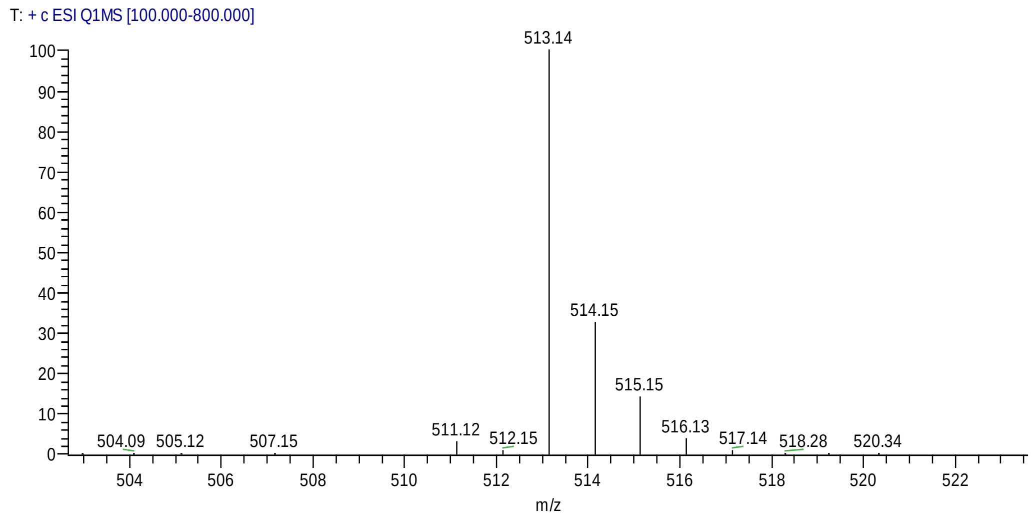

Figure S22. ESI-MS spectrum of the target compound **6a**

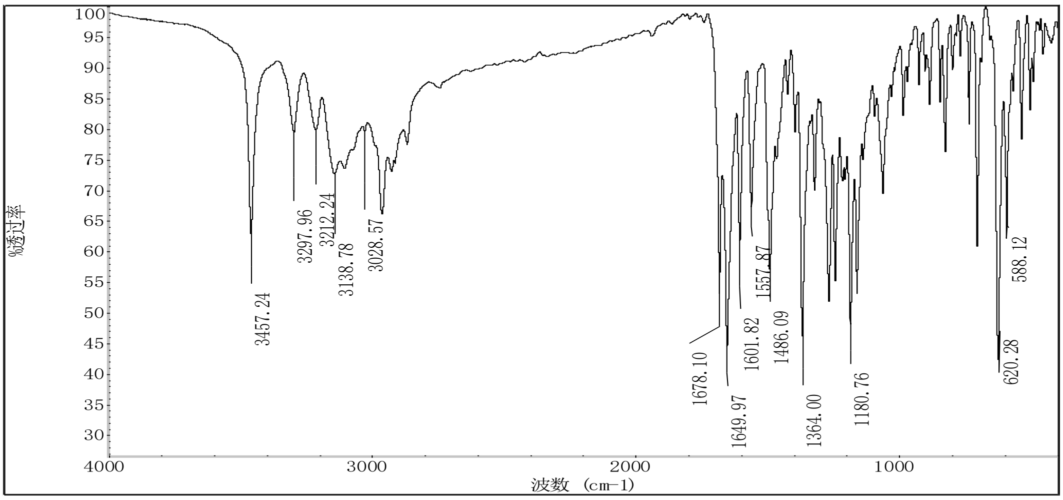

Figure S23. FT-IR spectrum of the target compound **6b**

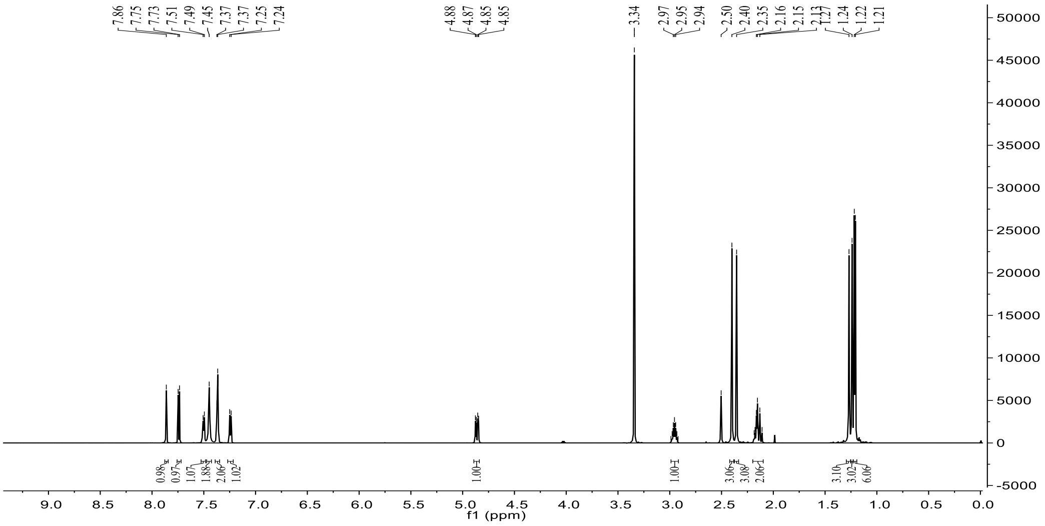

Figure S24. ^{1}H-NMR spectrum of the target compound **6b** in DMSO-d_6

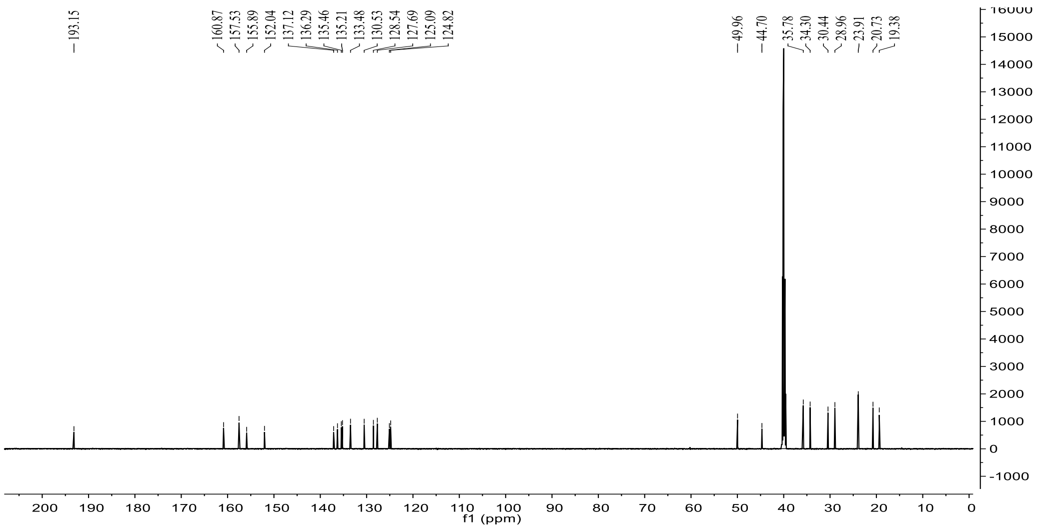

Figure S25. ^{13}C-NMR spectrum of the target compound **6b** in DMSO-d_6

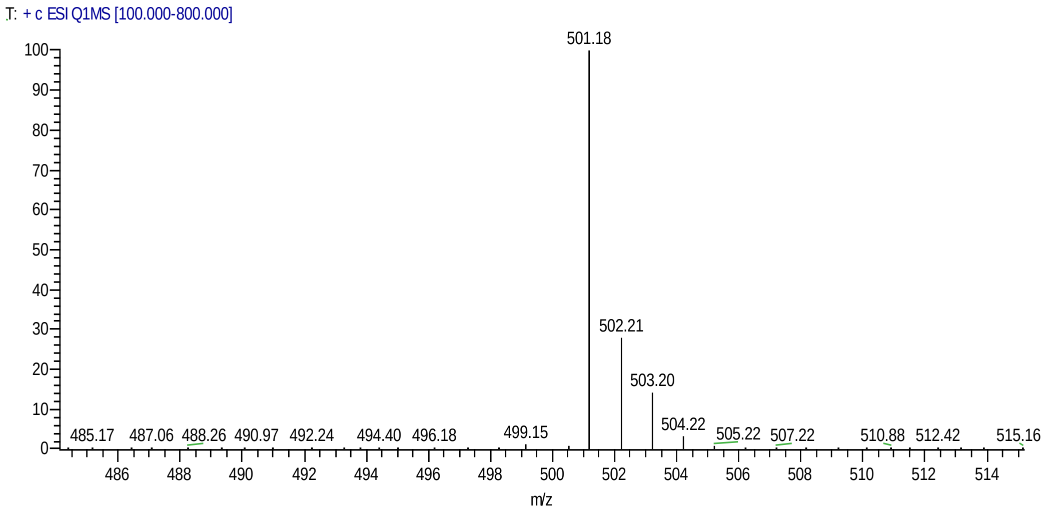

Figure S26. ESI-MS spectrum of the target compound **6b**

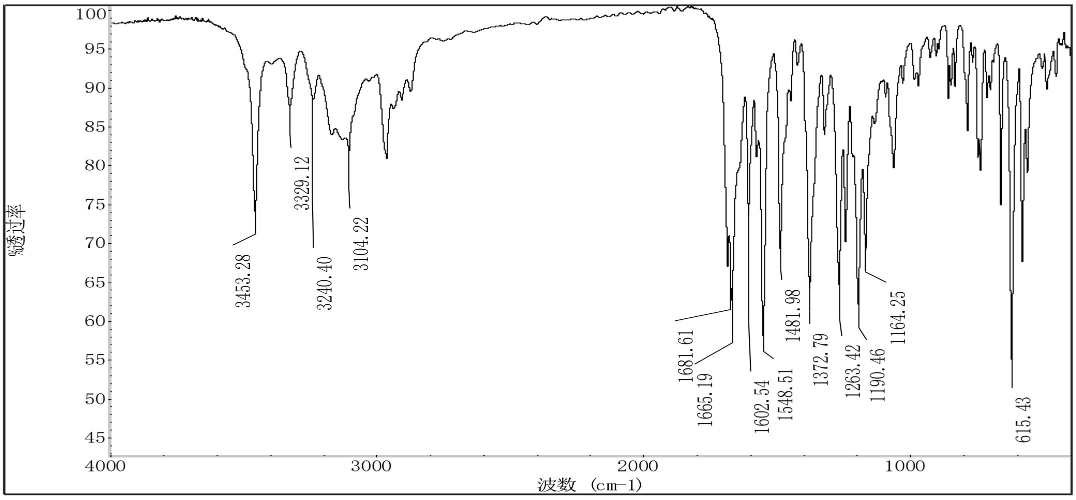

Figure S27. FT-IR spectru m of the target compound **6c**

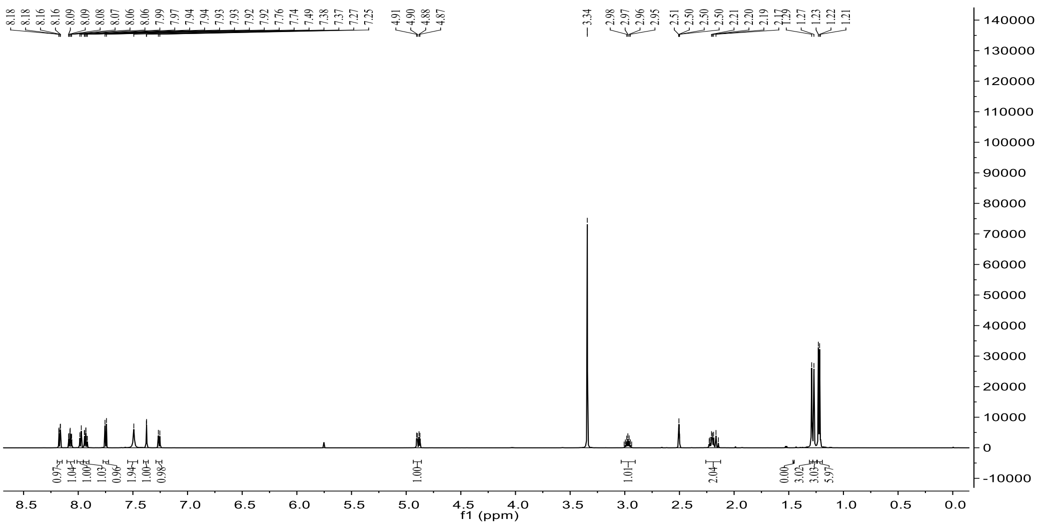

Figure S28. ^{1}H-NMR spectrum of the target compound **6c** in DMSO-d_6

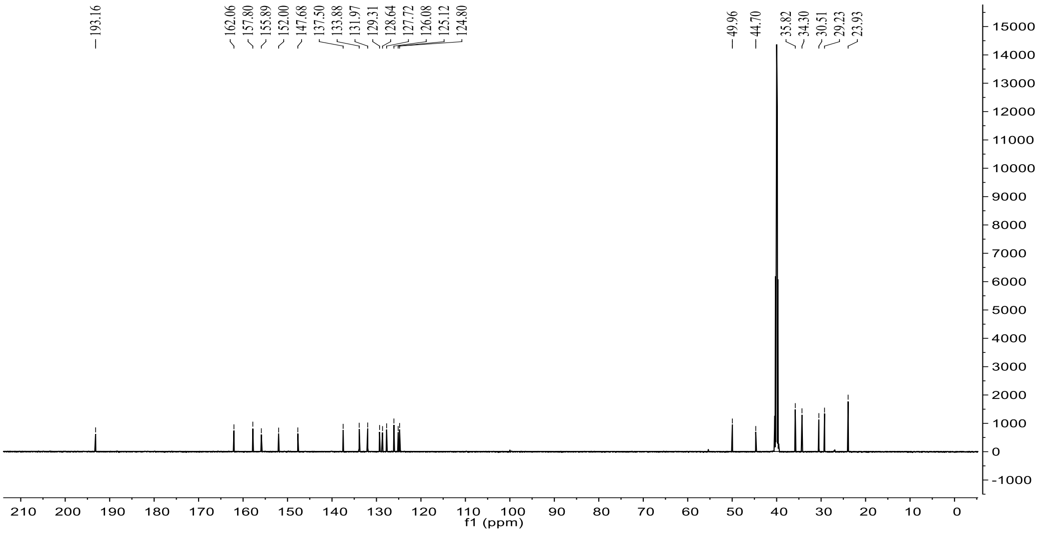

Figure S29. ^{13}C-NMR spectrum of the target compound **6c** in DMSO-d_6

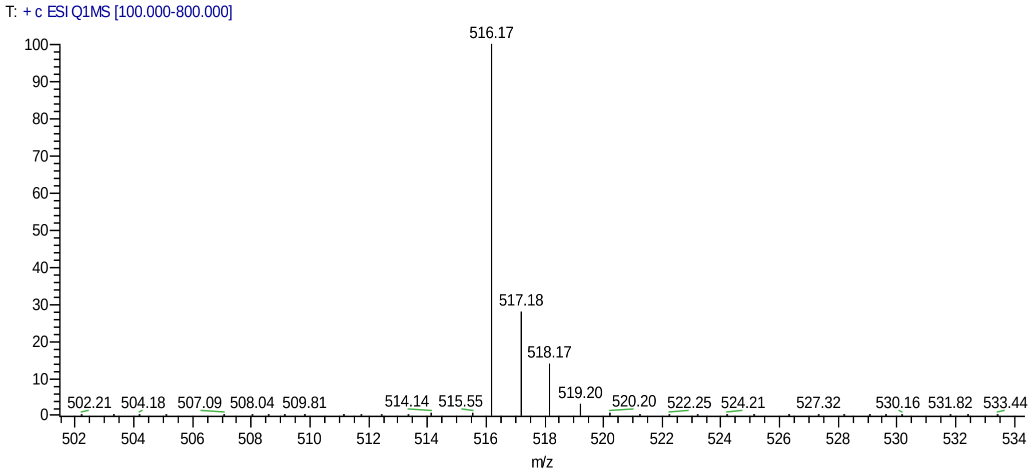

Figure S30. ESI-MS spectrum of the target compound **6c**

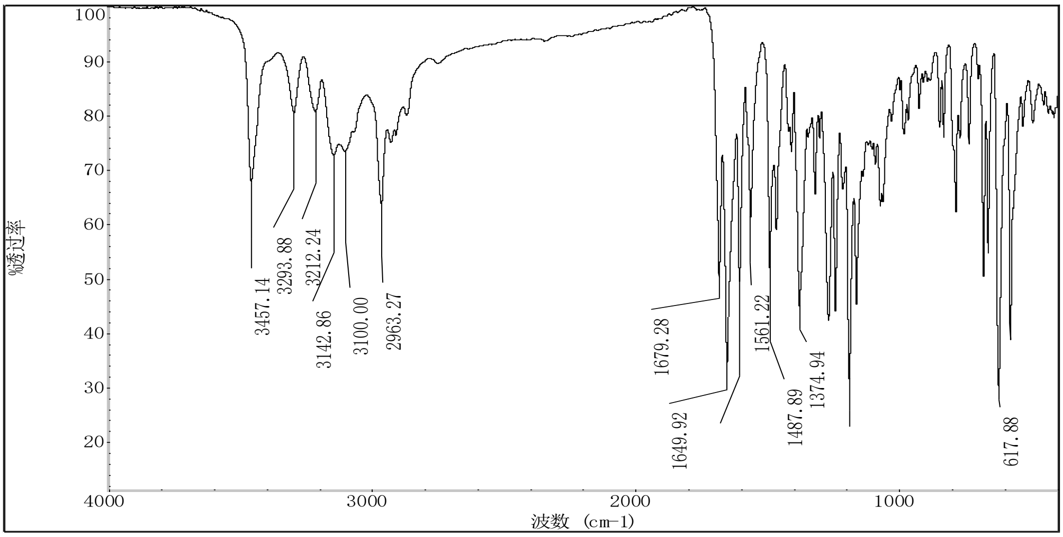

Figure S31. FT-IR spectrum of the target compound **6d**

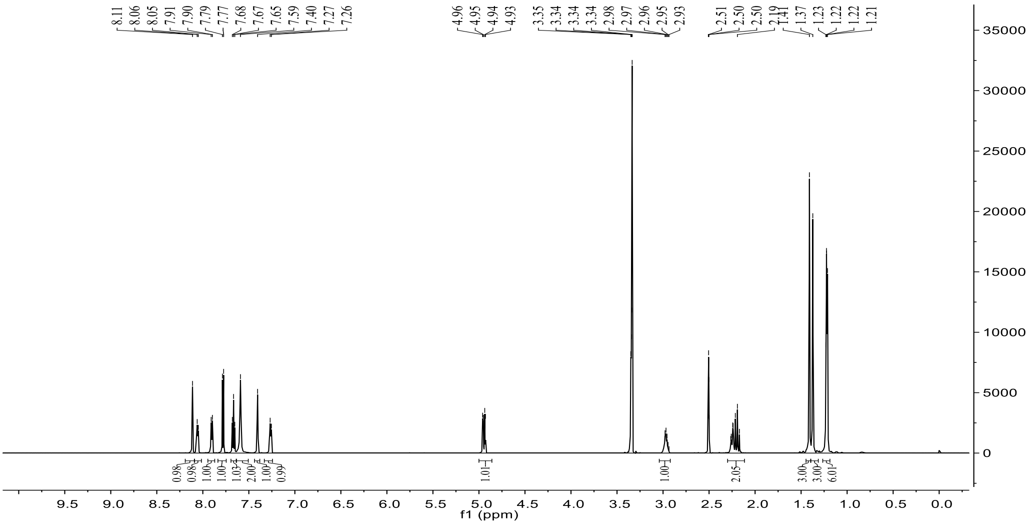

Figure S32. ^{1}H-NMR spectrum of the target compound **6d** in DMSO-d_6

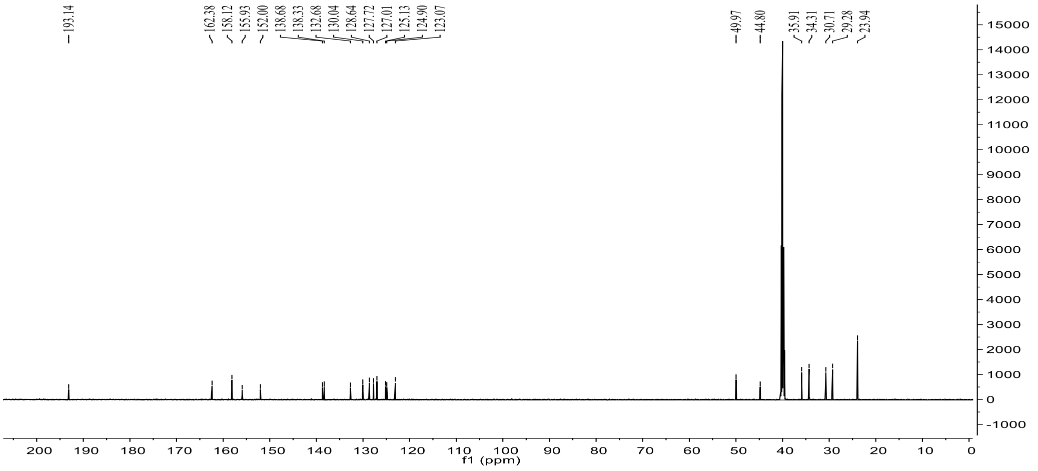

Figure S33. ^{13}C-NMR spectrum of the target compound **6d** in DMSO-d_6

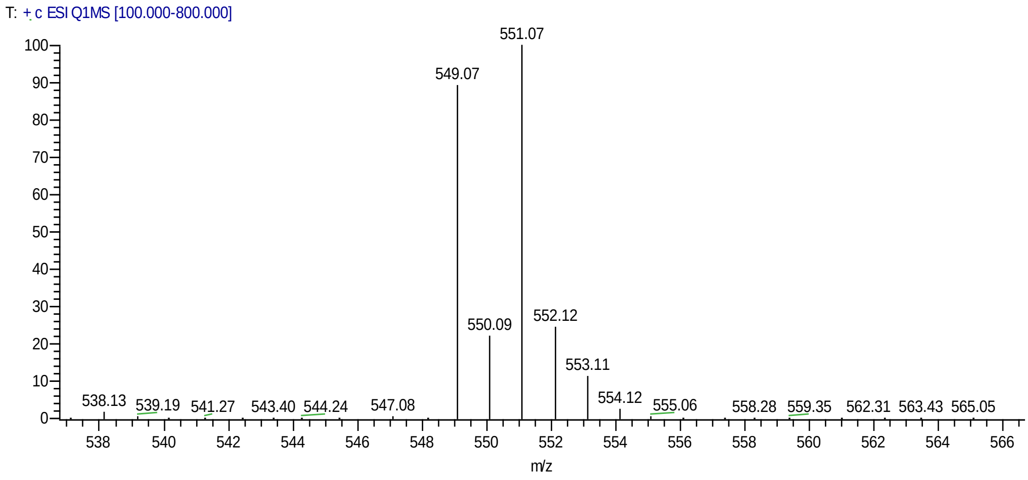

Figure S34. ESI-MS spectrum of the target compound **6d**

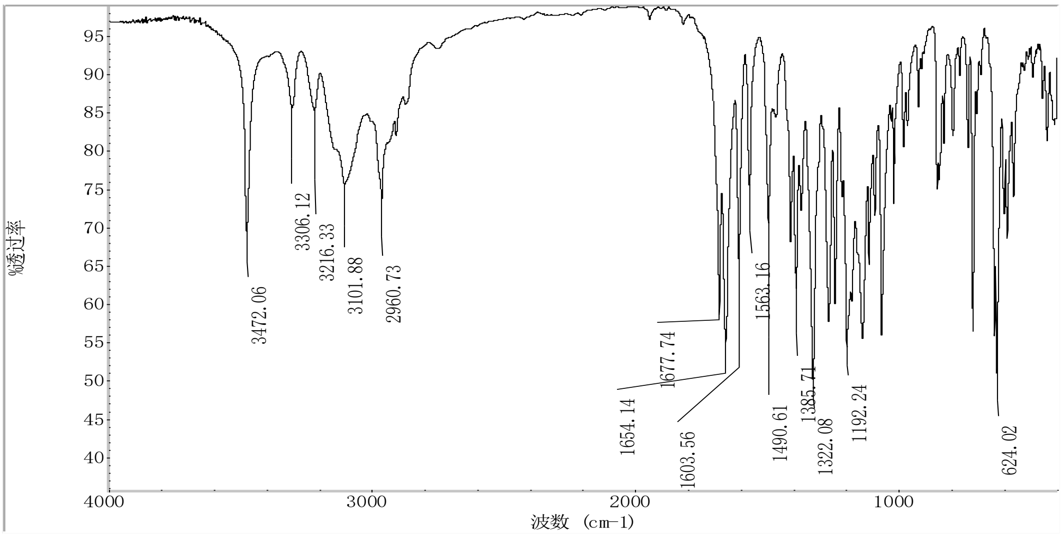

Figure S35. FT-IR spectrum of the target compound **6e**

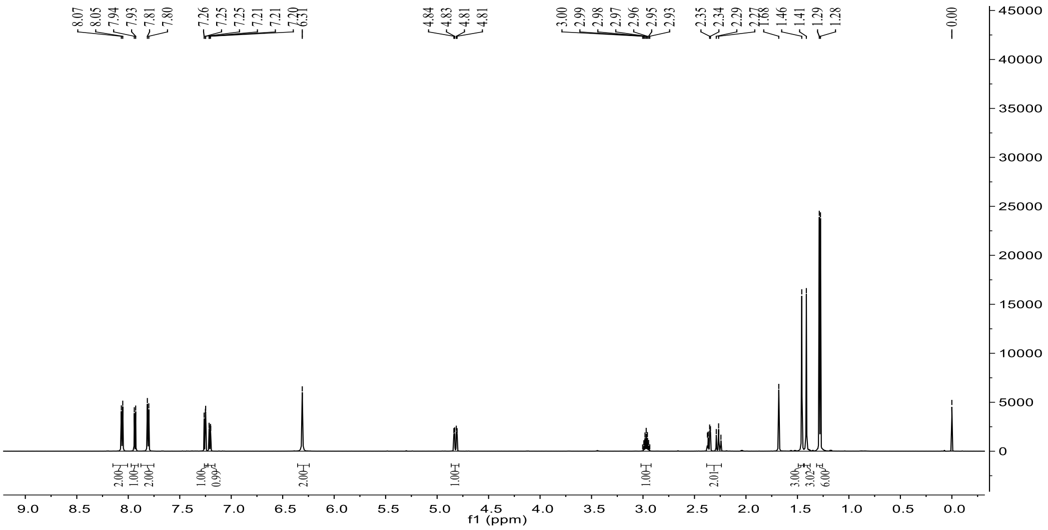

Figure S36. ^{1}H-NMR spectrum of the target compound **6e** in $CDCl_3$

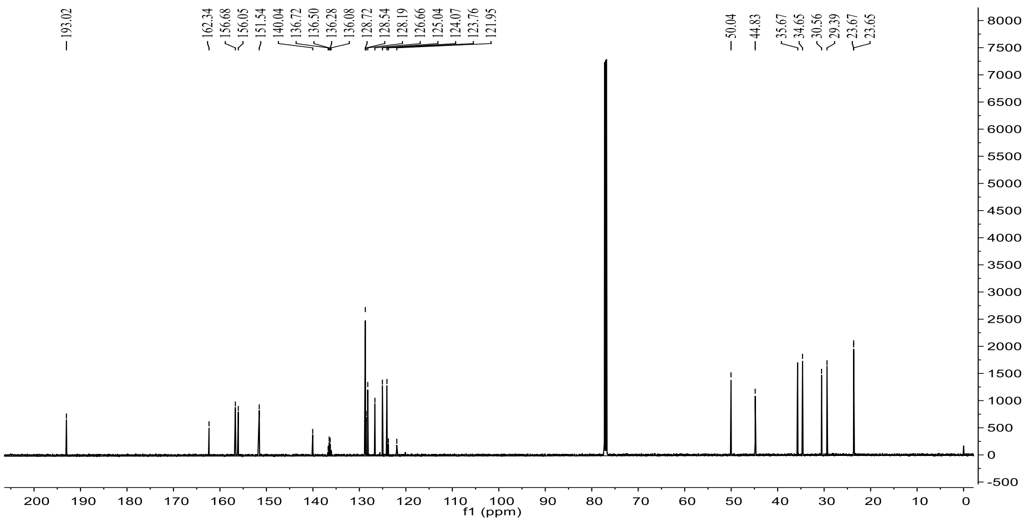

Figure S37. ^{13}C-NMR spectrum of the target compound **6e** in $CDCl_3$

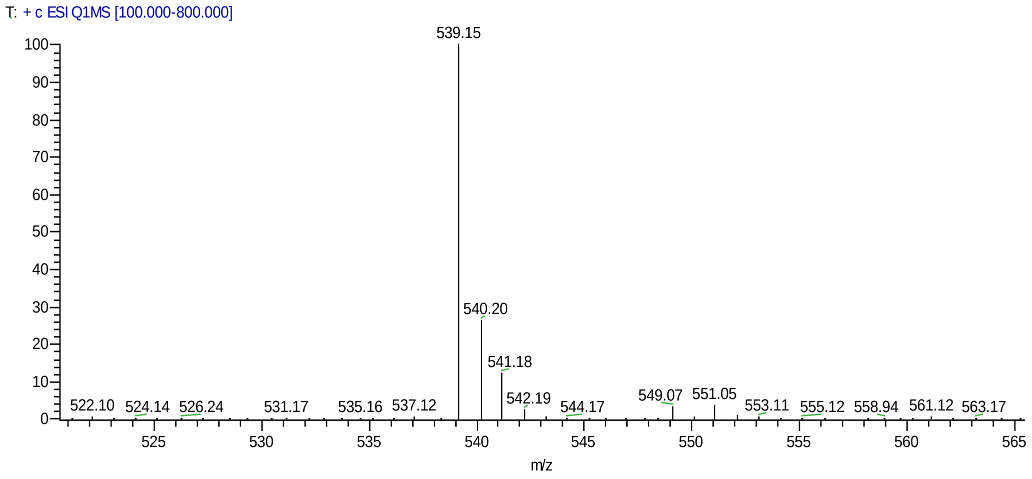

Figure S38. ESI-MS spectrum of the target compound **6e**

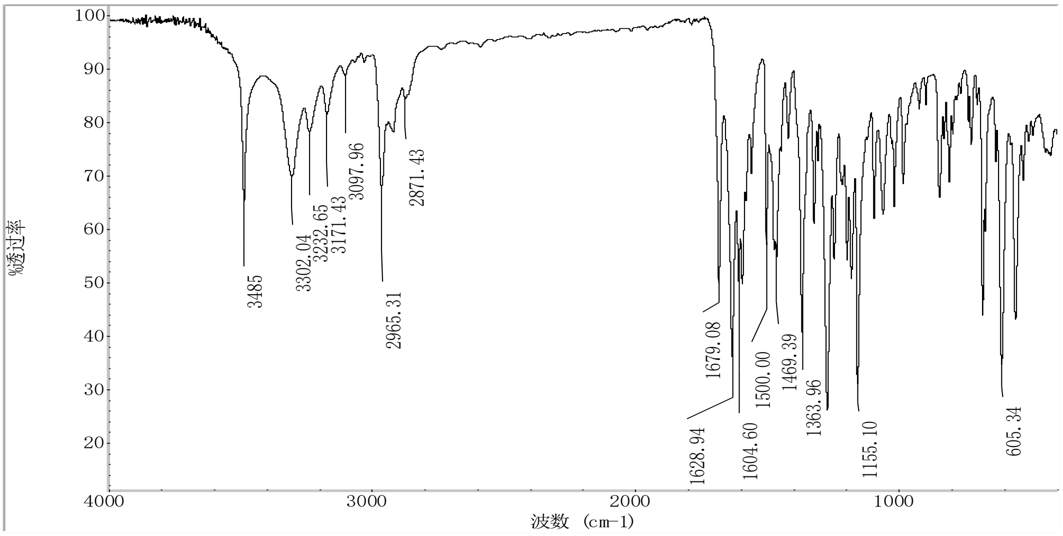

Figure S39. FT-IR spectrum of the target compound **6f**

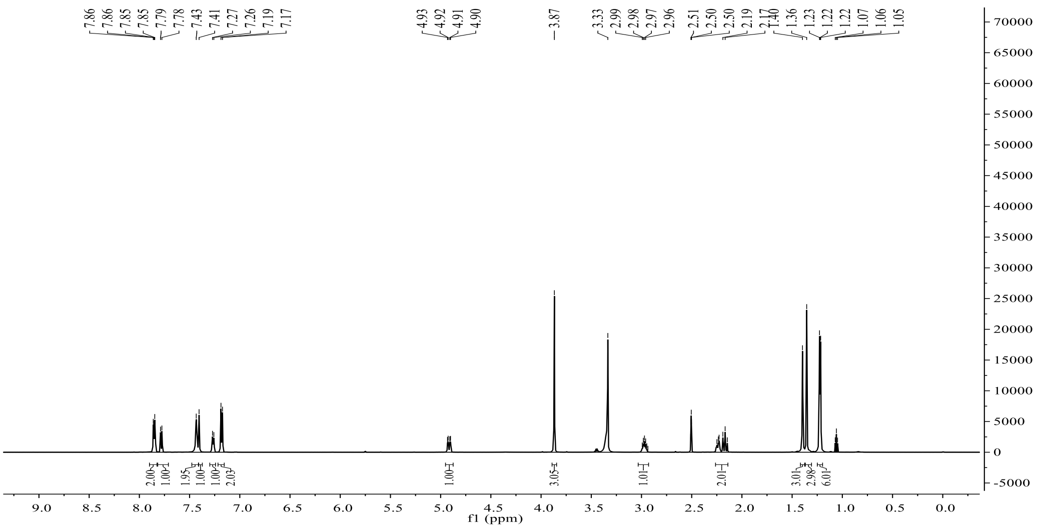

Figure S40. ^{1}H-NMR spectrum of the target compound **6f** in DMSO-d_6

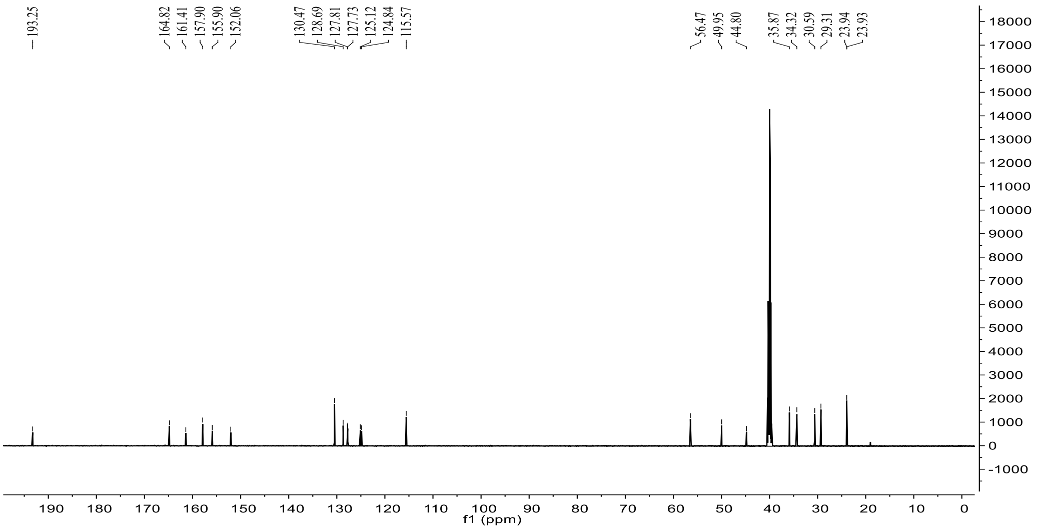

Figure S41. ^{13}C-NMR spectrum of the target compound **6f** in DMSO-d_6

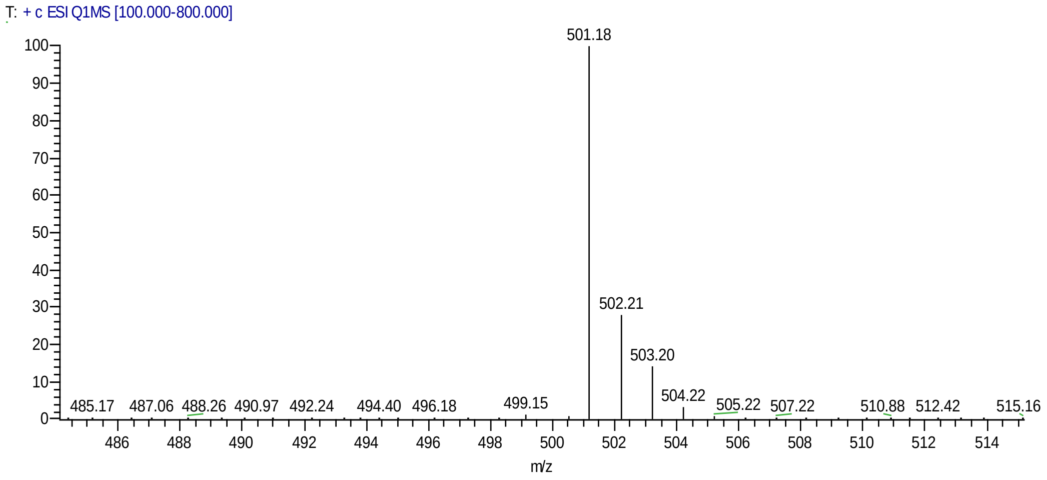

Figure S42. ESI-MS spectrum of the target compound **6f**

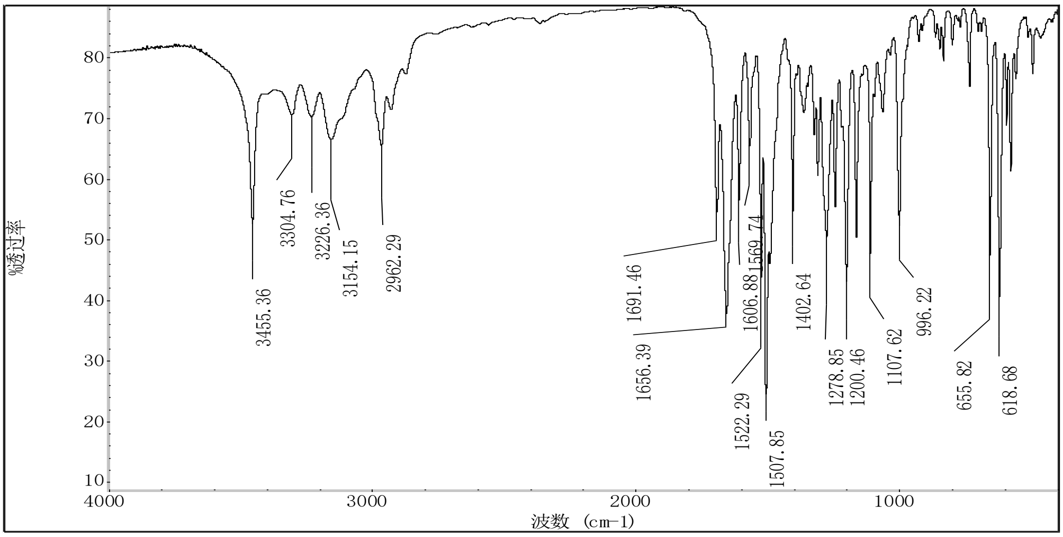

Figure S43. FT-IR spectrum of the target compound **6g**

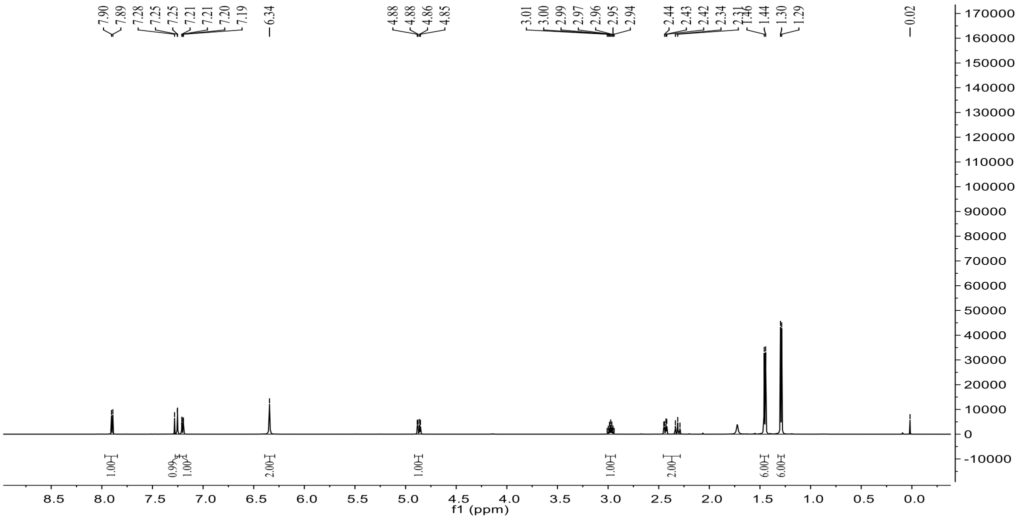

Figure S44. ^{1}H-NMR spectrum of the target compound **6g** in $CDCl_3$

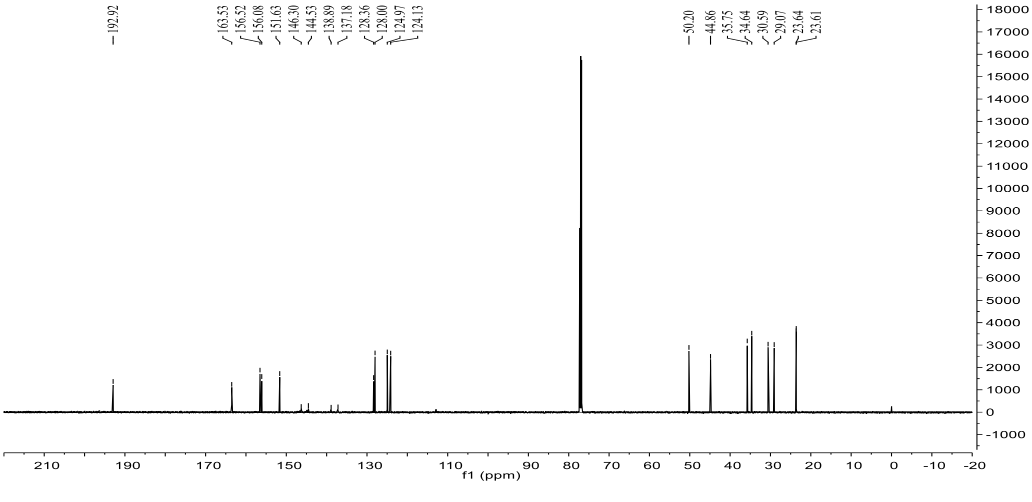

Figure S45. ^{13}C-NMR spectrum of the target compound **6g** in $CDCl_3$

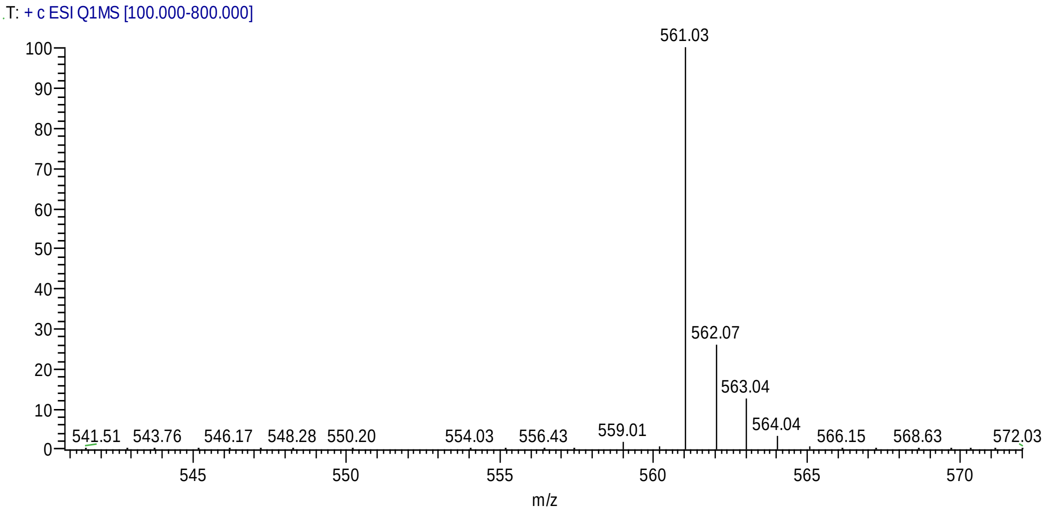

Figure S46. ESI-MS spectrum of the target compound **6g**

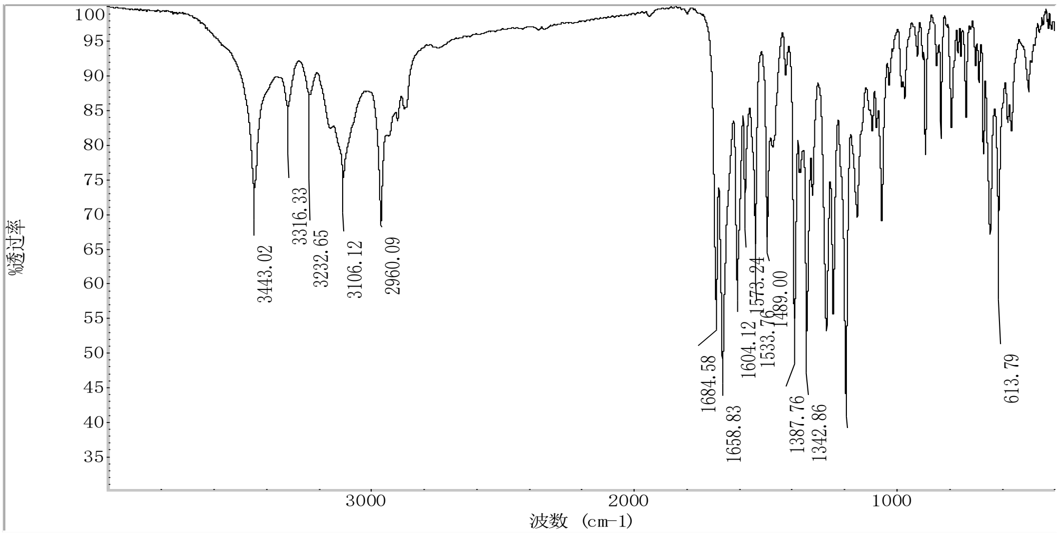

Figure S47. FT-IR spectrum of the target compound **6h**

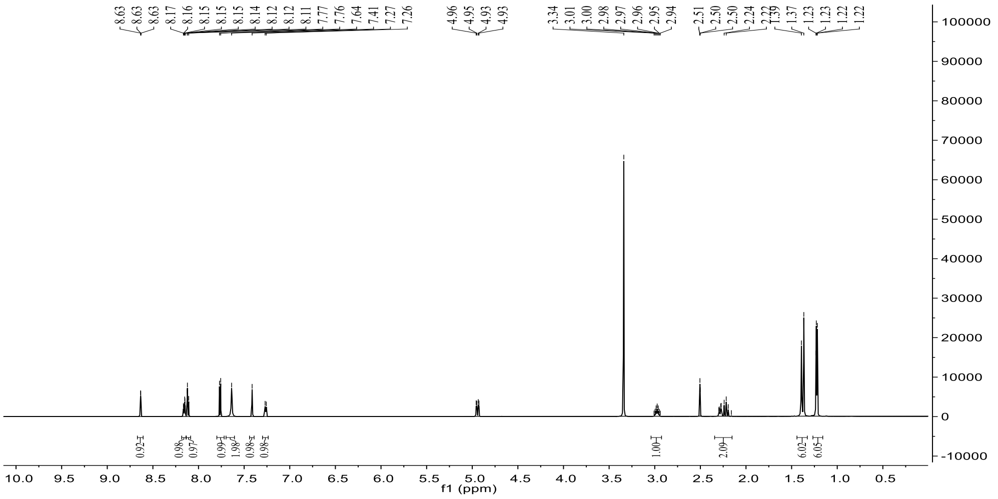

Figure S48. ^{1}H-NMR spectrum of the target compound **6h** in DMSO-d_6

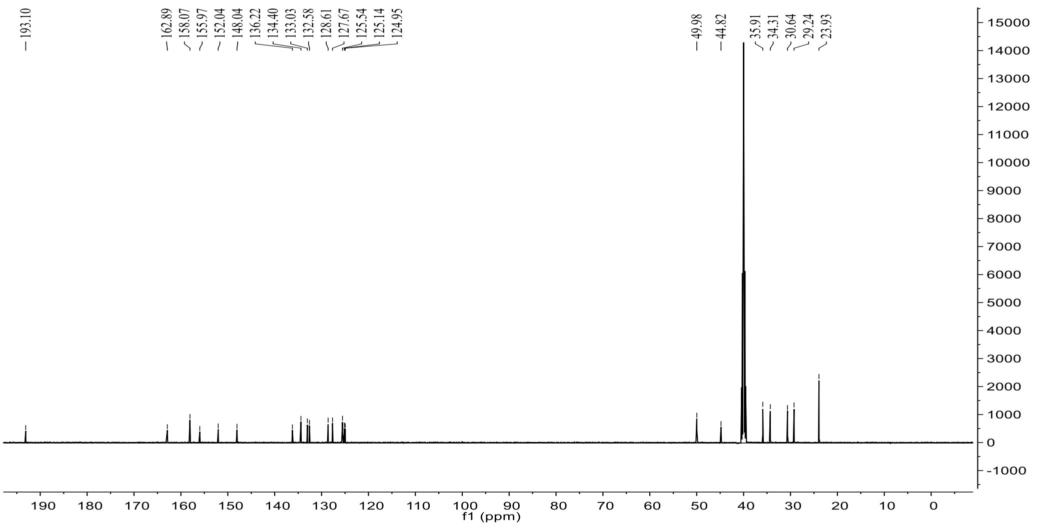

Figure S49. ^{13}C-NMR spectrum of the target compound **6h** in DMSO-d_6

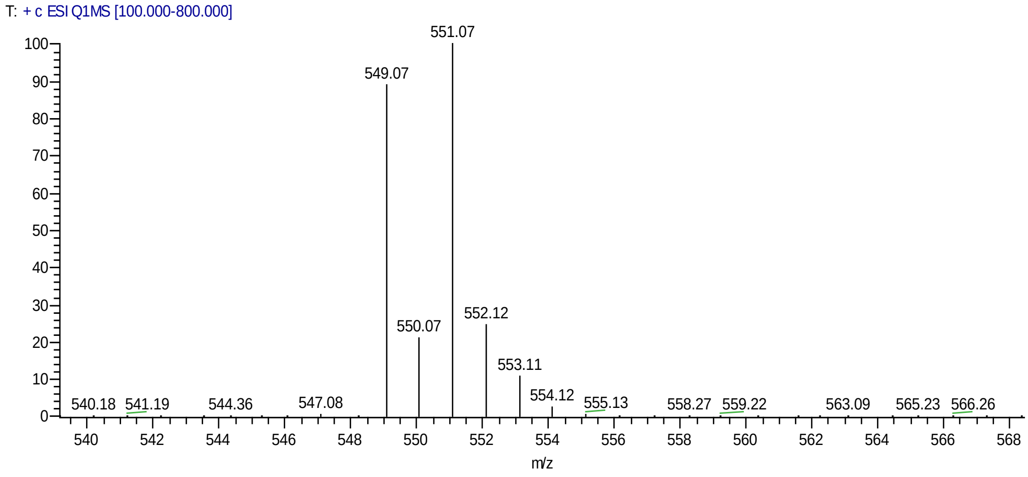

Figure S50. ESI-MS spectrum of the target compound **6h**

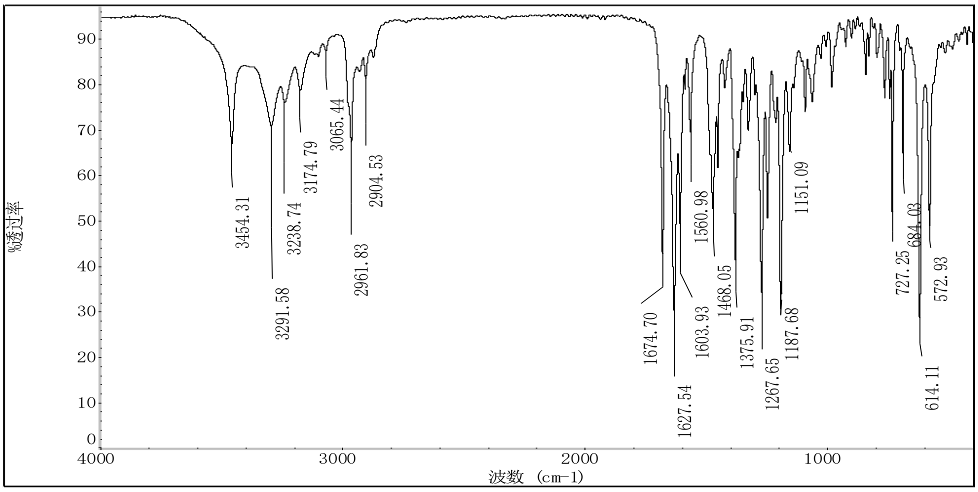

Figure S51. FT-IR spectrum of the target compound **6i**

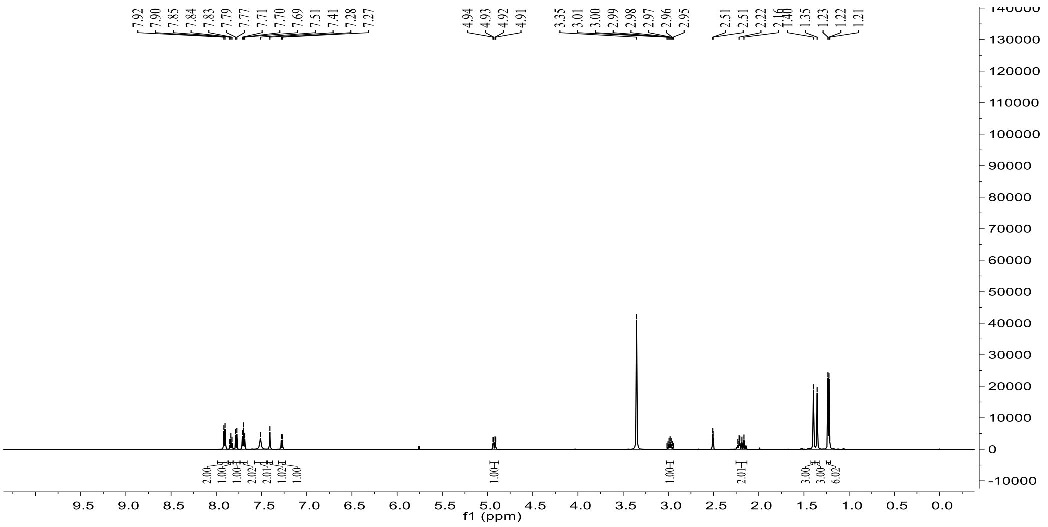

Figure S52. ^{1}H-NMR spectrum of the target compound **6i** in DMSO-d_6

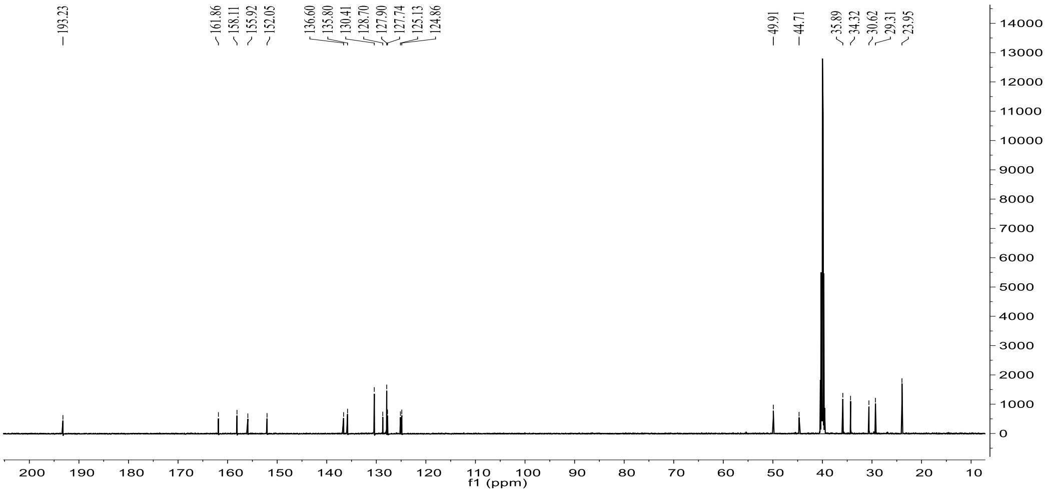

Figure S53. ^{13}C-NMR spectrum of the target compound **6i** in DMSO-d_6

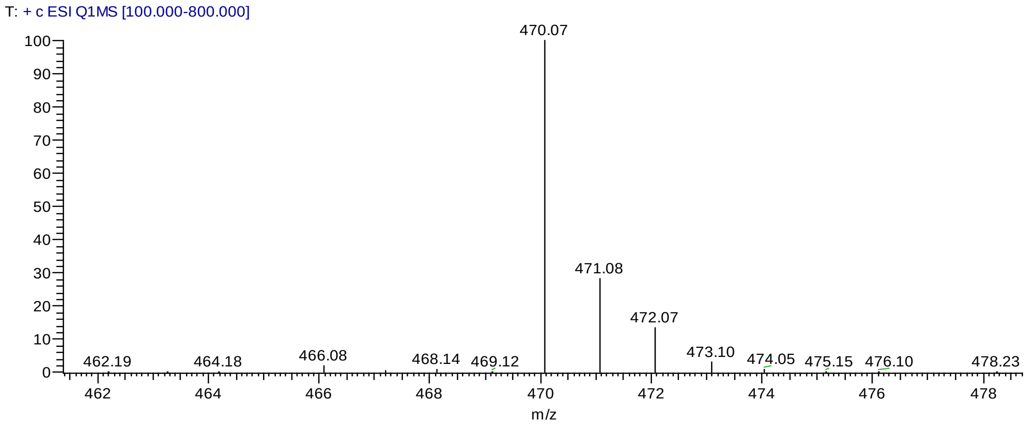

Figure S54. ESI-MS spectrum of the target compound **6i**

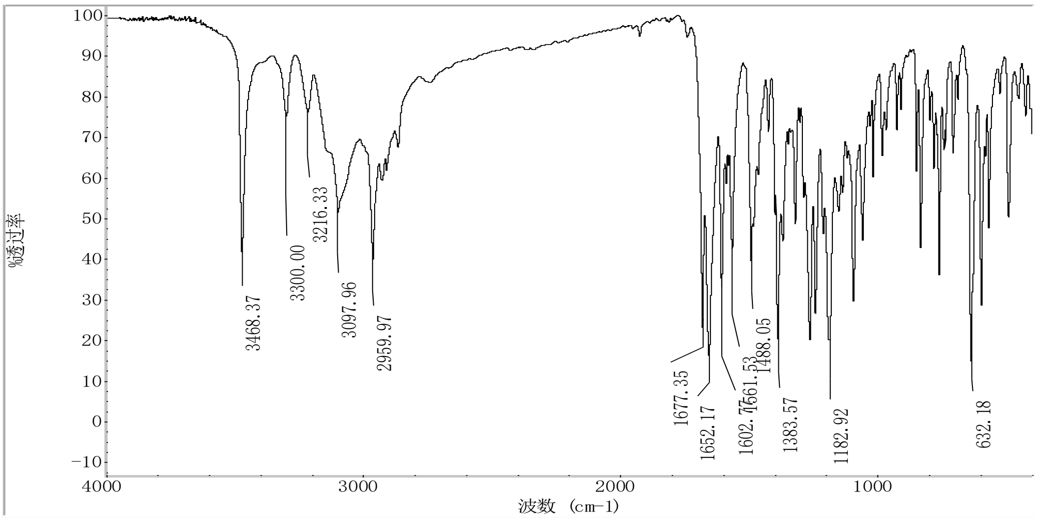

Figure S55. FT-IR spectrum of the target compound **6j**

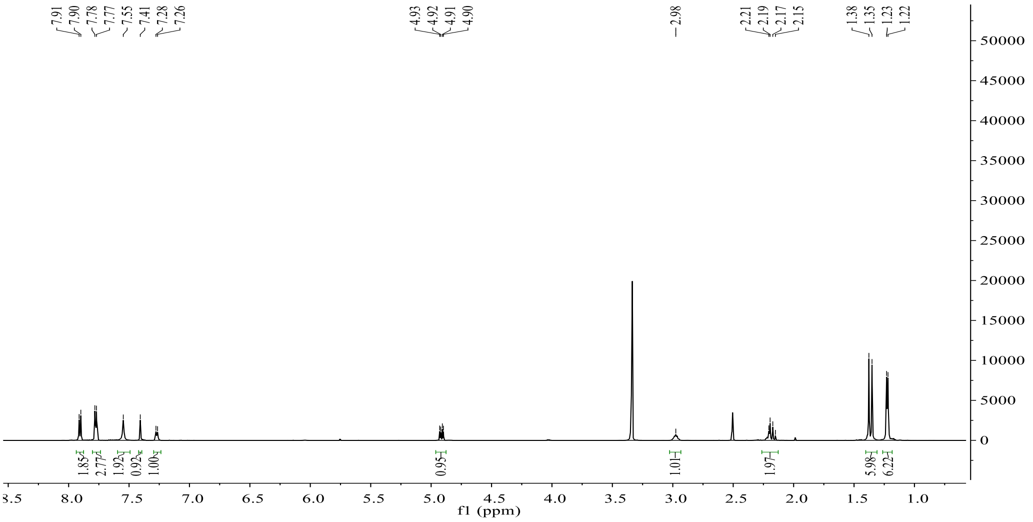

Figure S56. ^{1}H-NMR spectrum of the target compound **6j** in DMSO-d_6

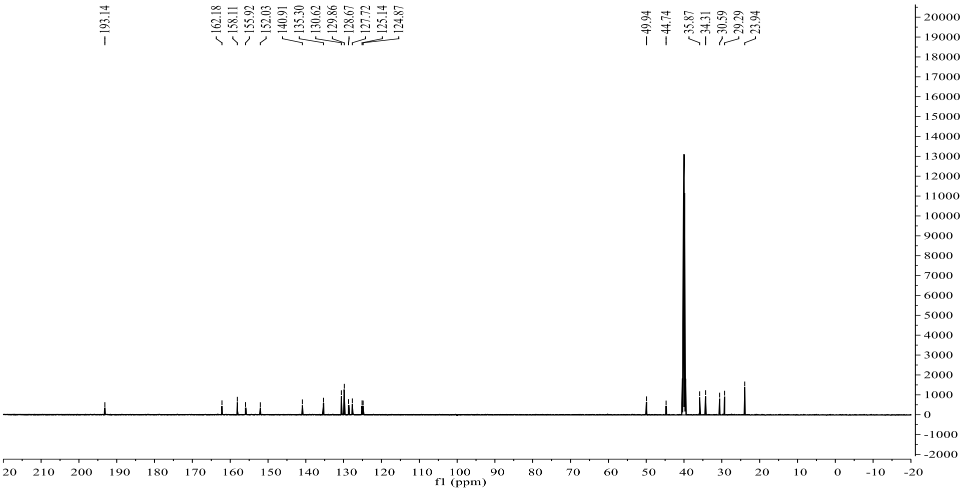

Figure S57. ^{13}C-NMR spectrum of the target compound **6j** in DMSO-d_6

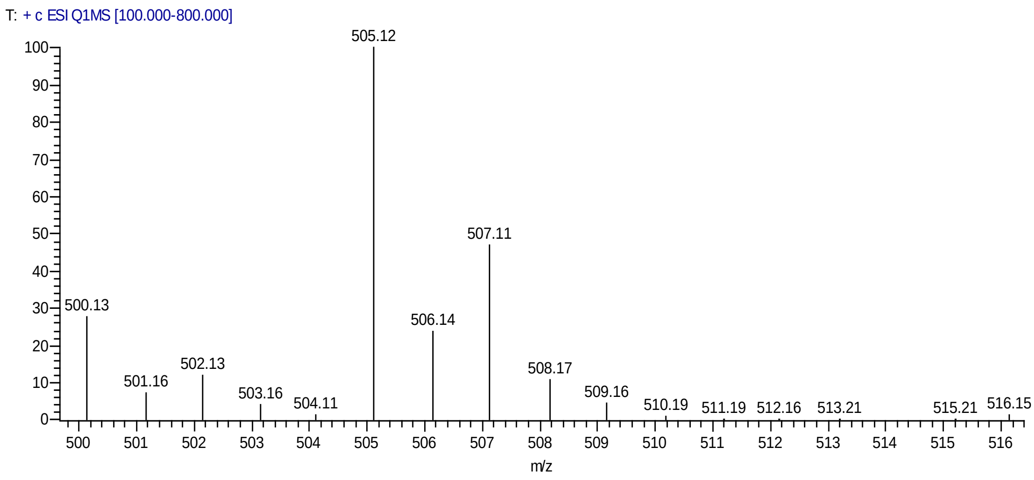

Figure S58. ESI-MS spectrum of the target compound **6j**

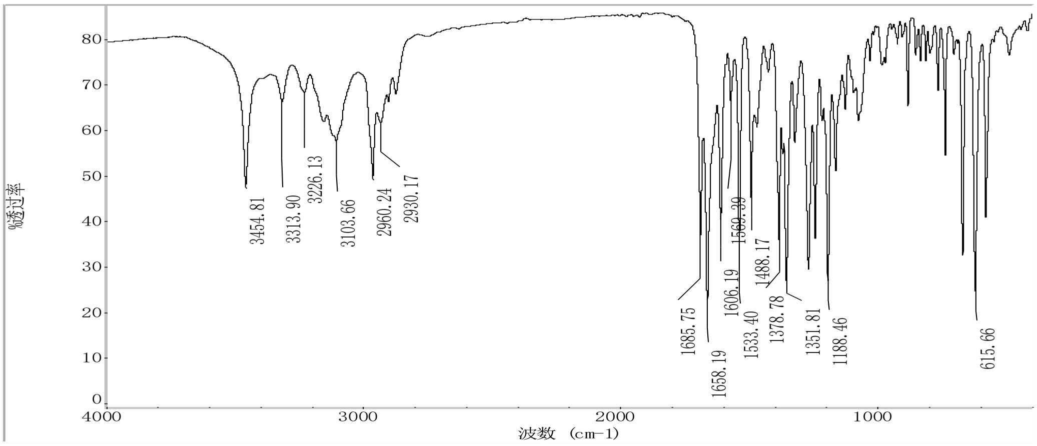

Figure S59. FT-IR spectrum of the target compound **6k**

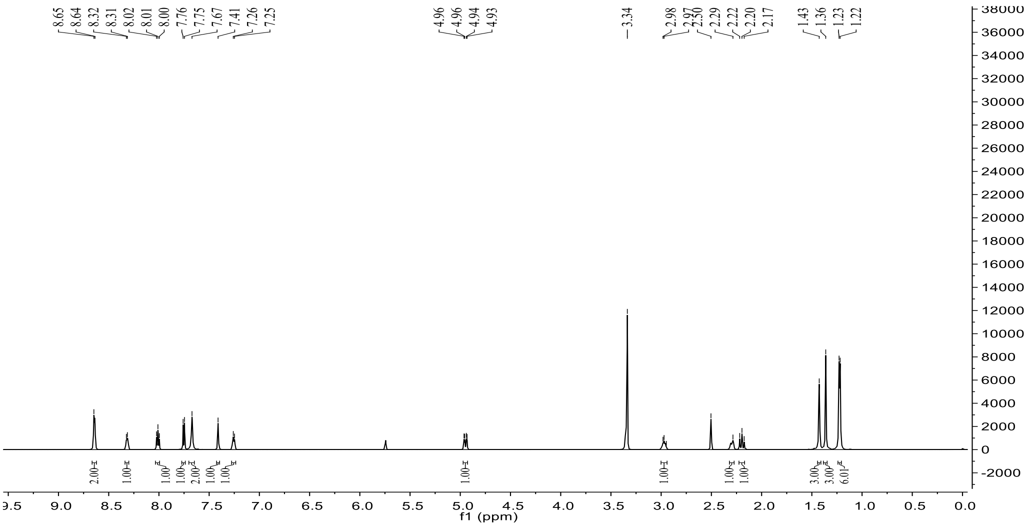

Figure S60. ^{1}H-NMR spectrum of the target compound **6k** in DMSO-d_6

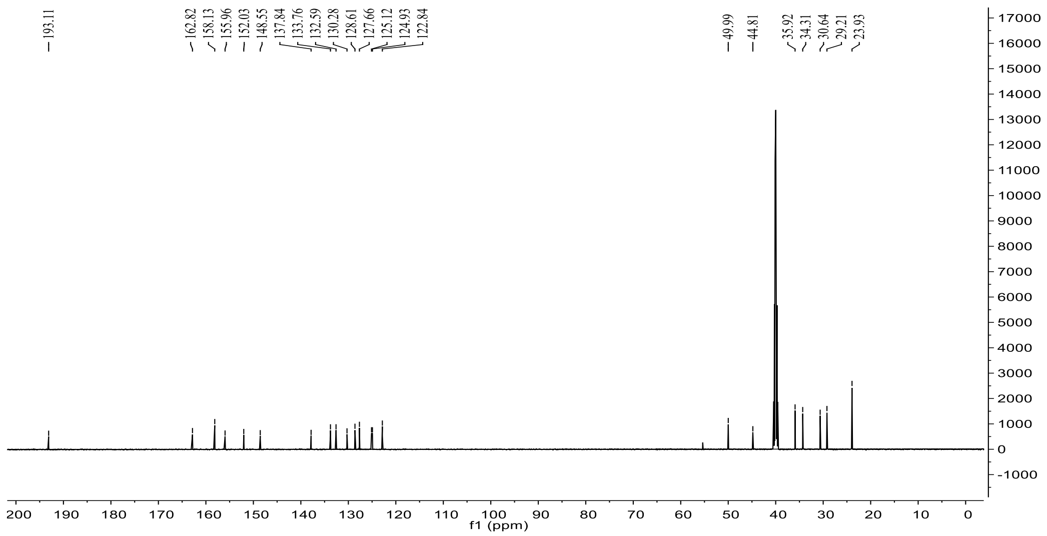

Figure S61. ^{13}C-NMR spectrum of the target compound **6k** in DMSO-d_6

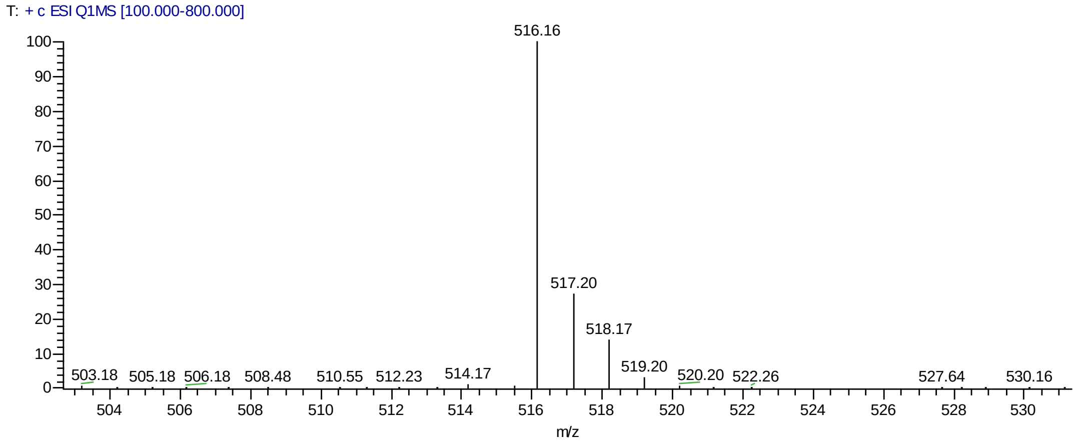

Figure S62. ESI-MS spectrum of the target compound **6k**

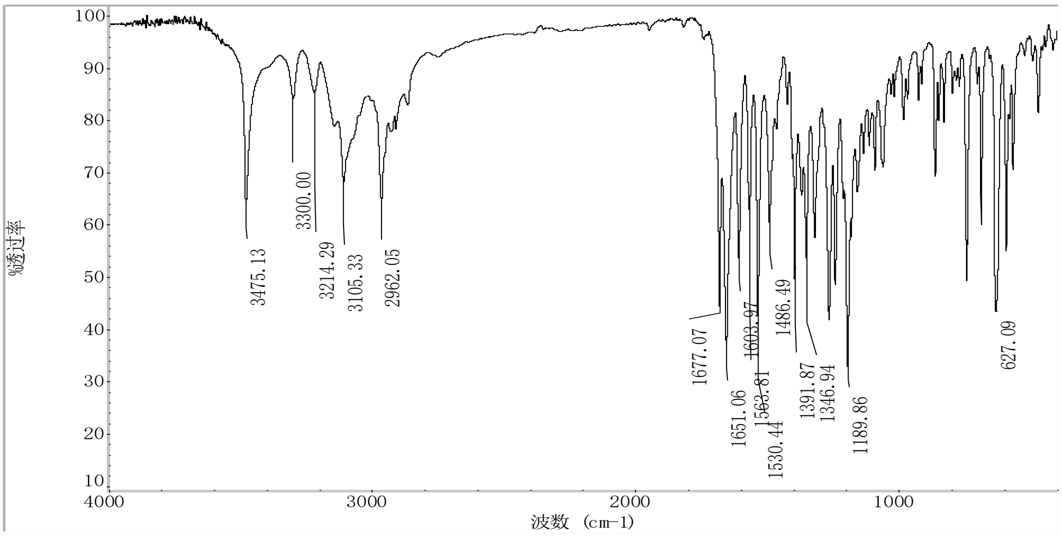

Figure S63. FT-IR spectrum of the target compound **6l**

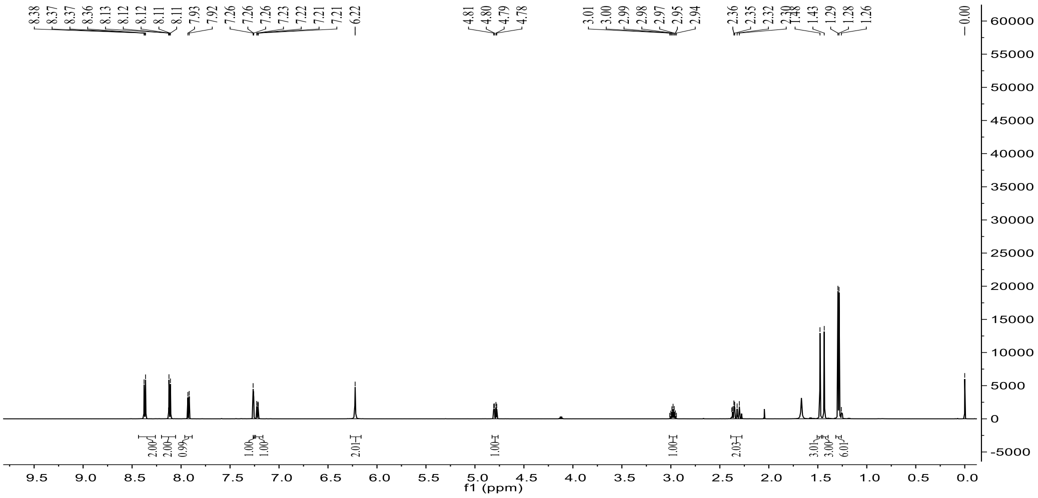

Figure S64. ^{1}H-NMR spectrum of the target compound **6l** in $CDCl_3$

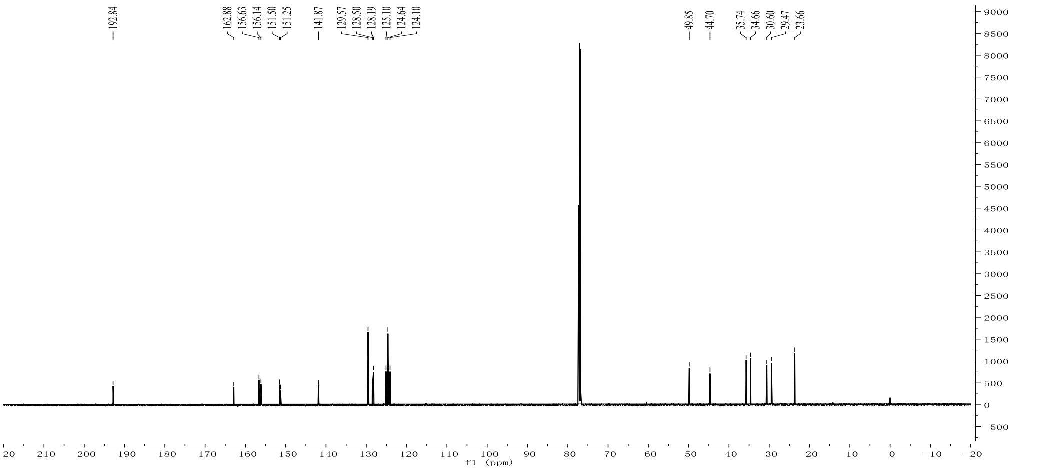

Figure S65. ^{13}C-NMR spectrum of the target compound **6l** in $CDCl_3$

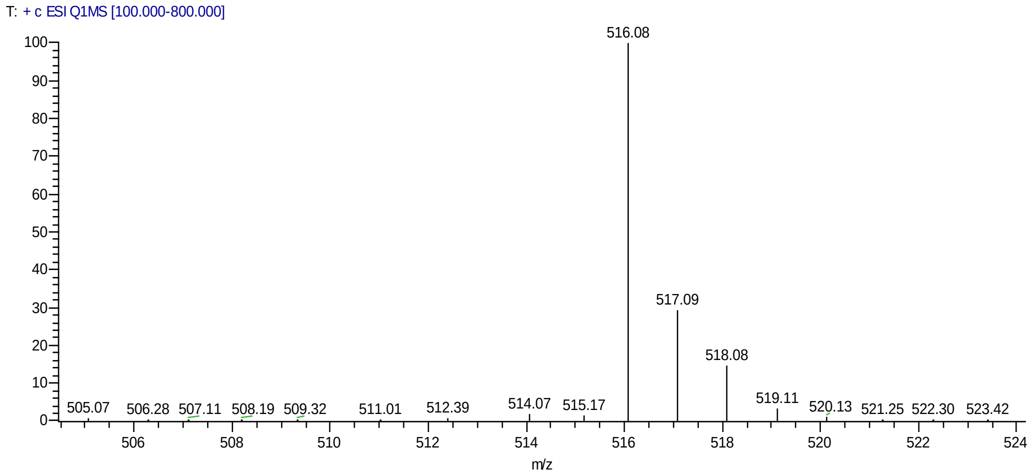

Figure S66. ESI-MS spectrum of the target compound **6l**

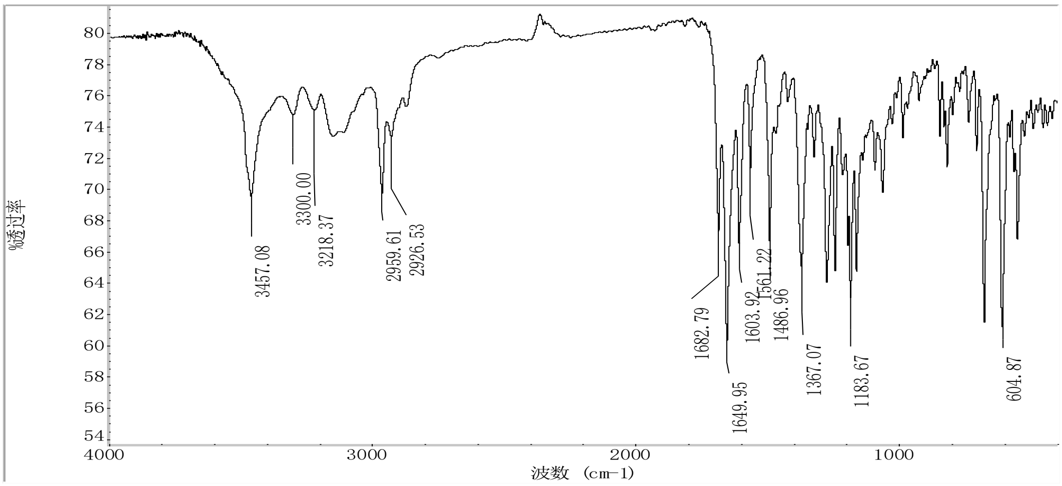

Figure S67. FT-IR spectrum of the target compound **6m**

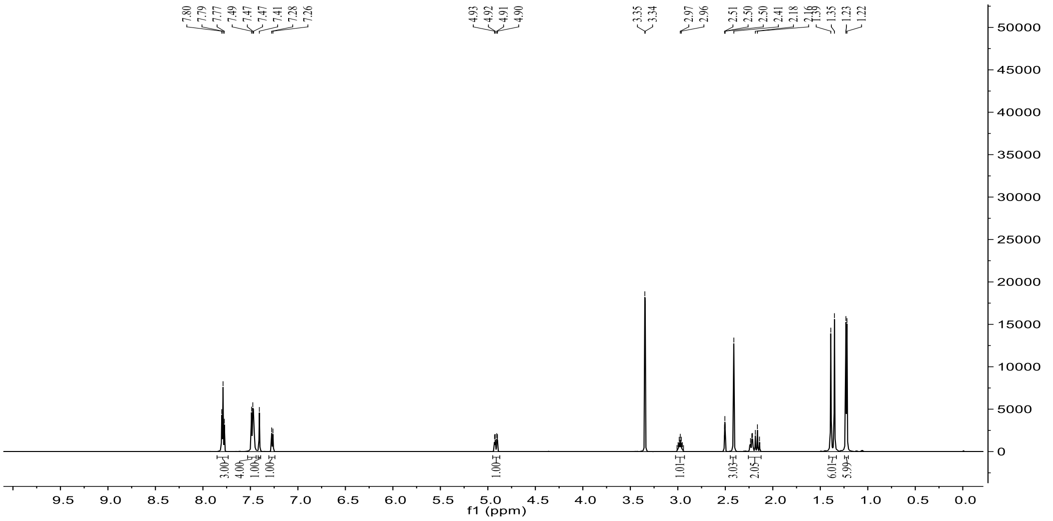

Figure S68. ^{1}H-NMR spectrum of the target compound **6m** in DMSO-d_6

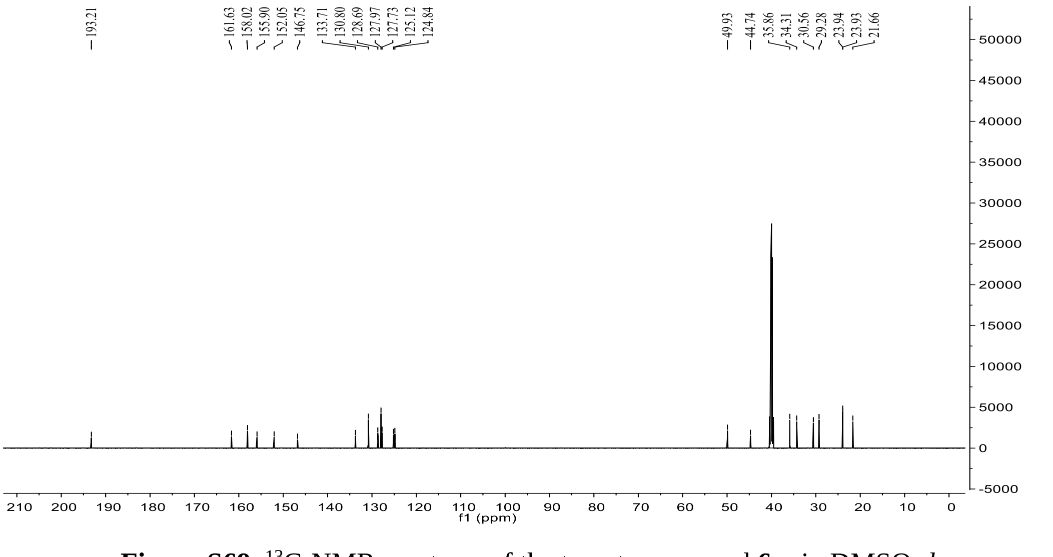

Figure S69. ^{13}C-NMR spectrum of the target compound **6m** in DMSO-d_6

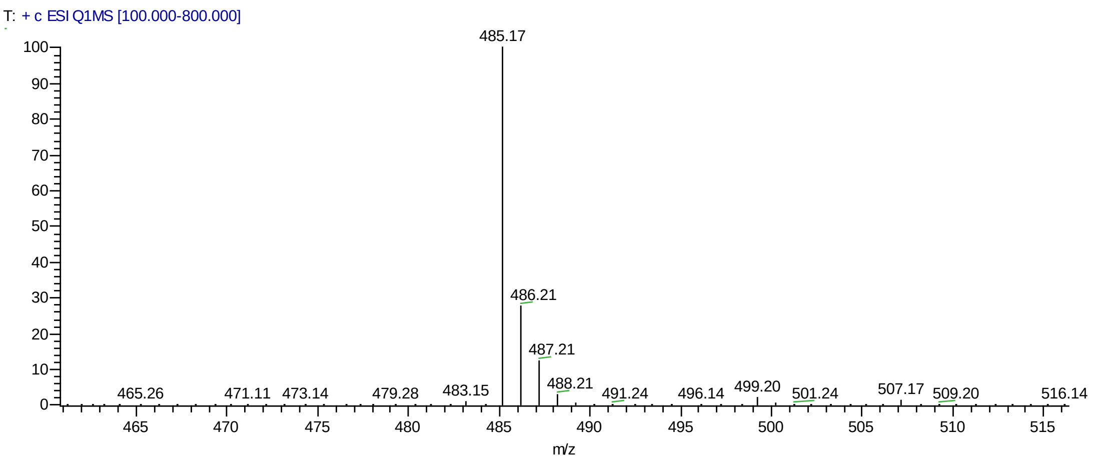

Figure S70. ESI-MS spectrum of the target compound **6m**

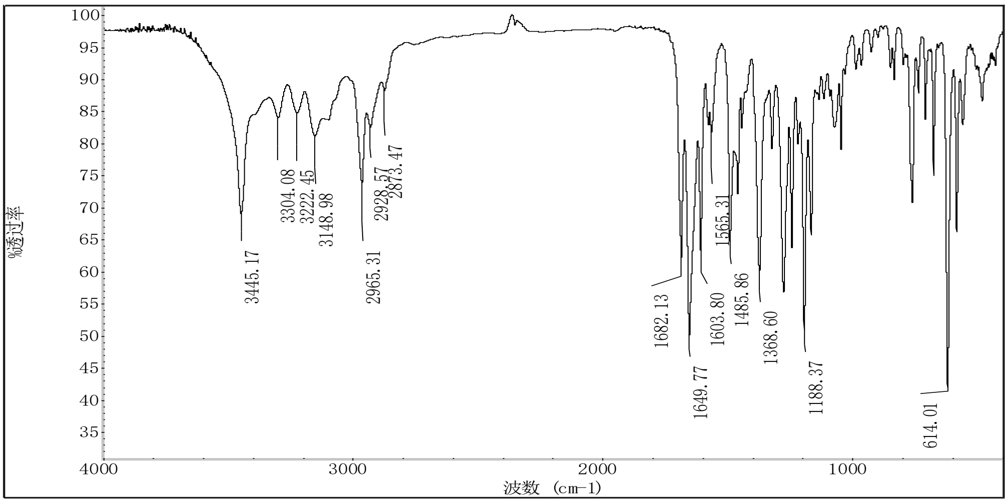

Figure S71. FT-IR spectrum of the target compound **6n**

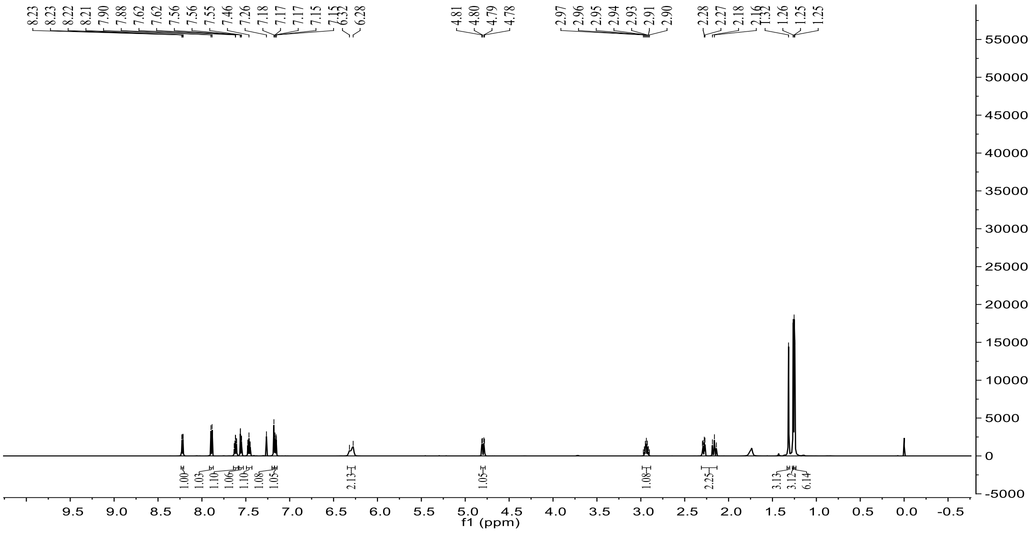

Figure S72. ^{1}H-NMR spectrum of the target compound **6n** in $CDCl_3$

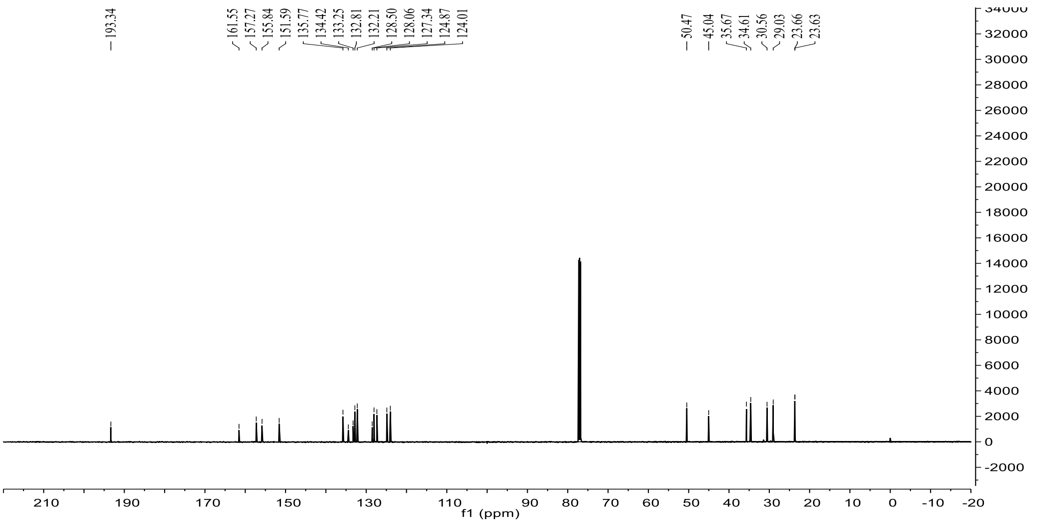

Figure S73. ^{13}C-NMR spectrum of the target compound **6n** in $CDCl_3$

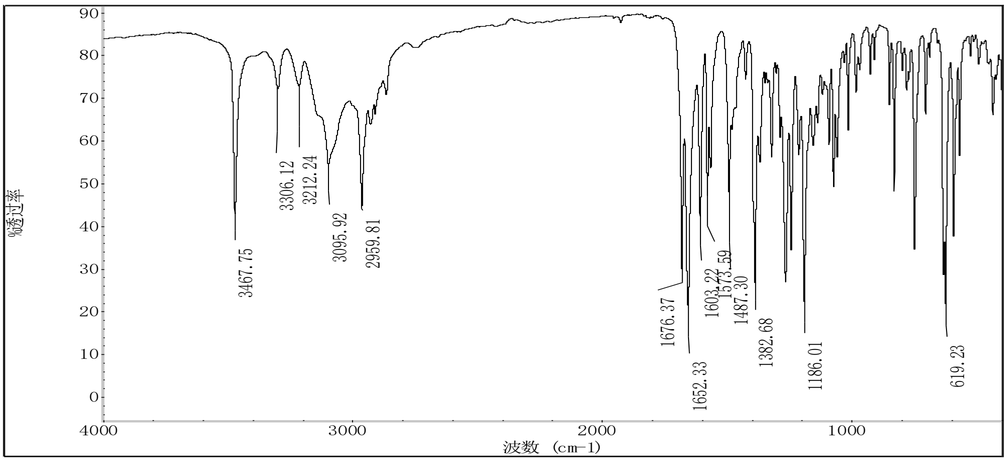

Figure S74. FT-IR spectrum of the target compound **6o**

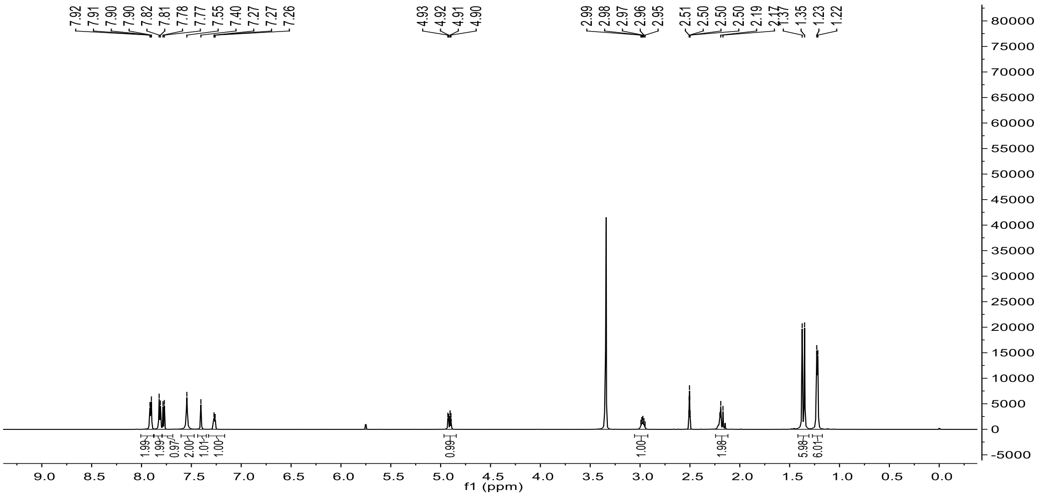

Figure S75. ^{1}H-NMR spectrum of the target compound **6o** in DMSO-d_6

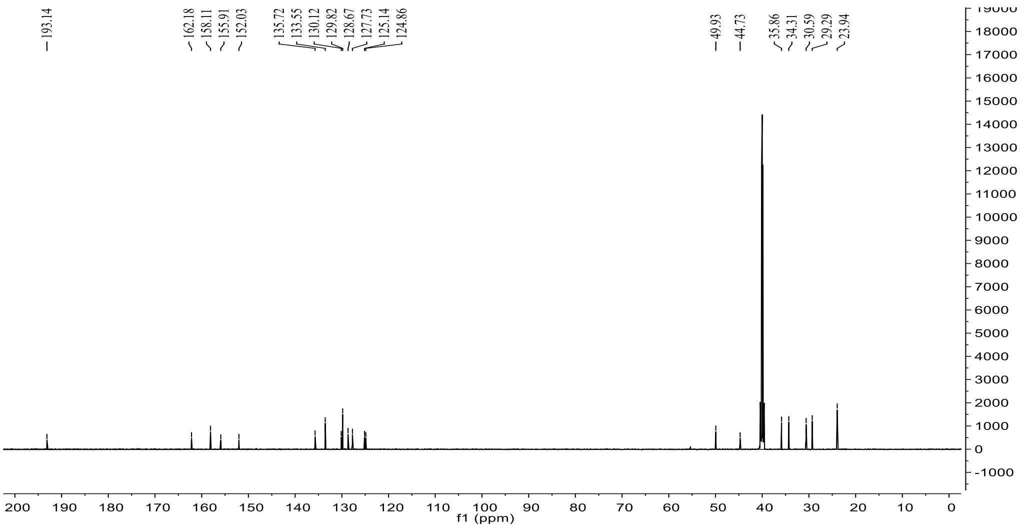

Figure S76. ^{13}C-NMR spectrum of the target compound **6o** in DMSO-d_6

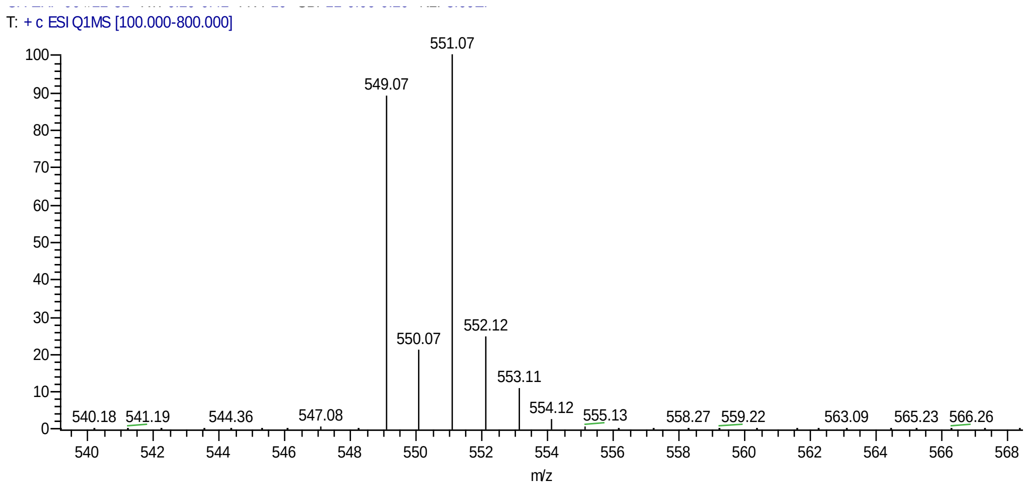

Figure S77. ESI-MS spectrum of the target compound **6o**

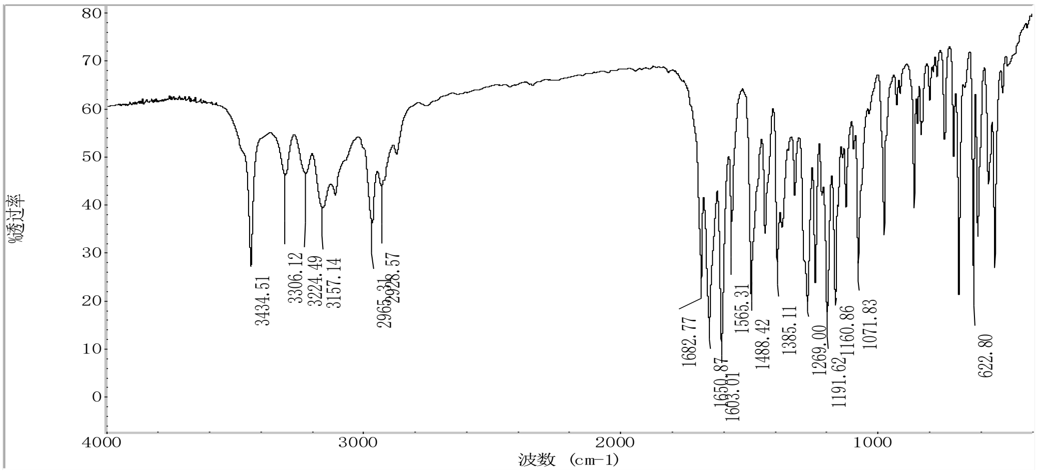

Figure S78. FT-IR spectrum of the target compound **6p**

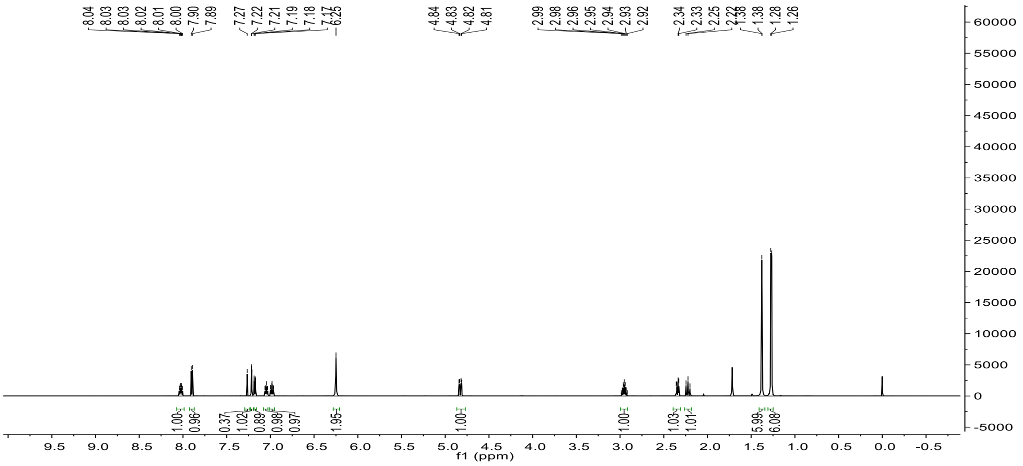

Figure S79. ^{1}H-NMR spectrum of the target compound **6p** in $CDCl_3$

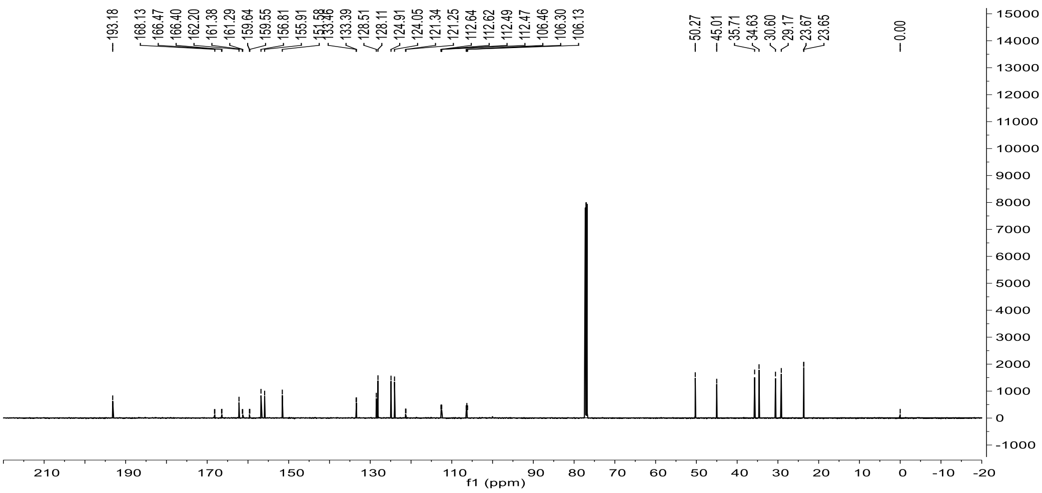

Figure S80. ^{13}C-NMR spectrum of the target compound **6p** in $CDCl_3$

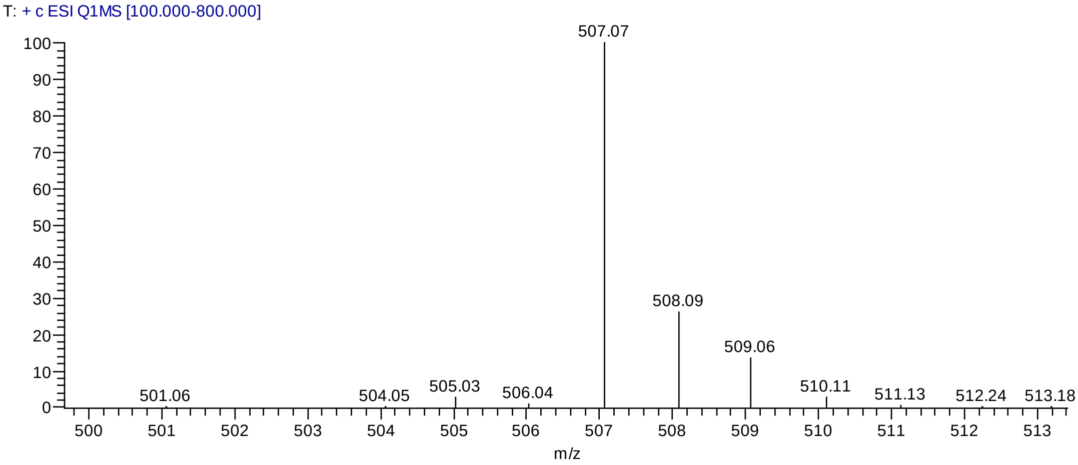

Figure S81. ESI-MS spectrum of the target compound **6p**

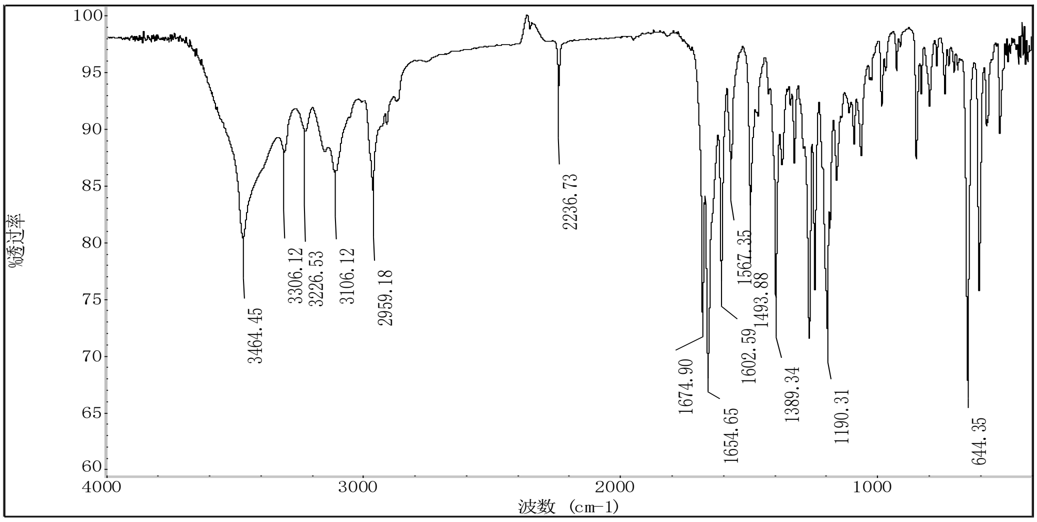

Figure S82. FT-IR spectrum of the target compound **6q**

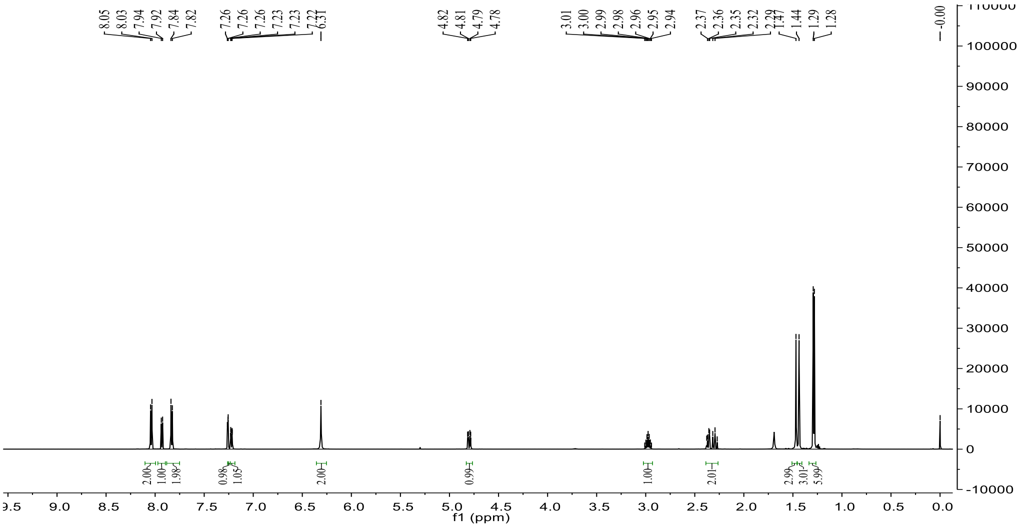

Figure S83. ^{1}H-NMR spectrum of the target compound **6q** in $CDCl_3$

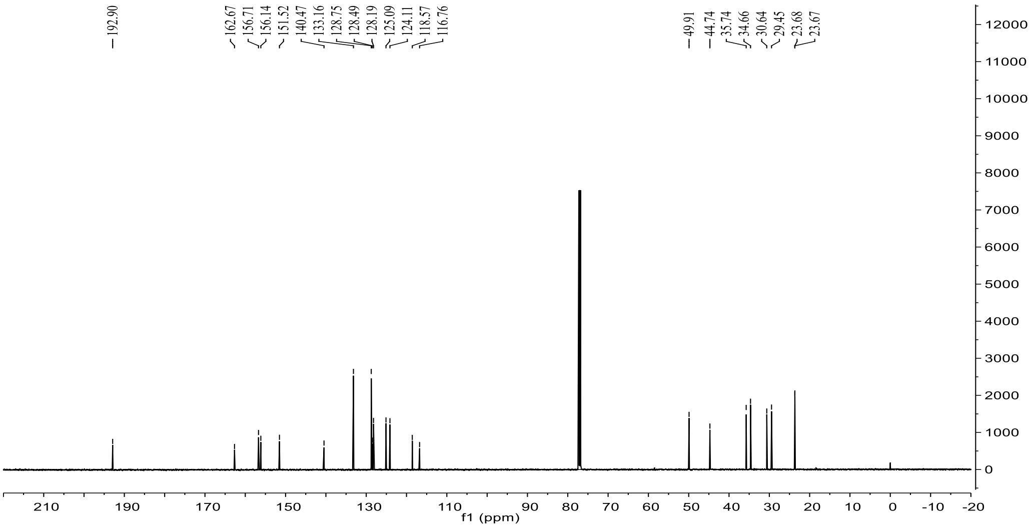

Figure S84. ^{13}C-NMR spectrum of the target compound **6q** in $CDCl_3$

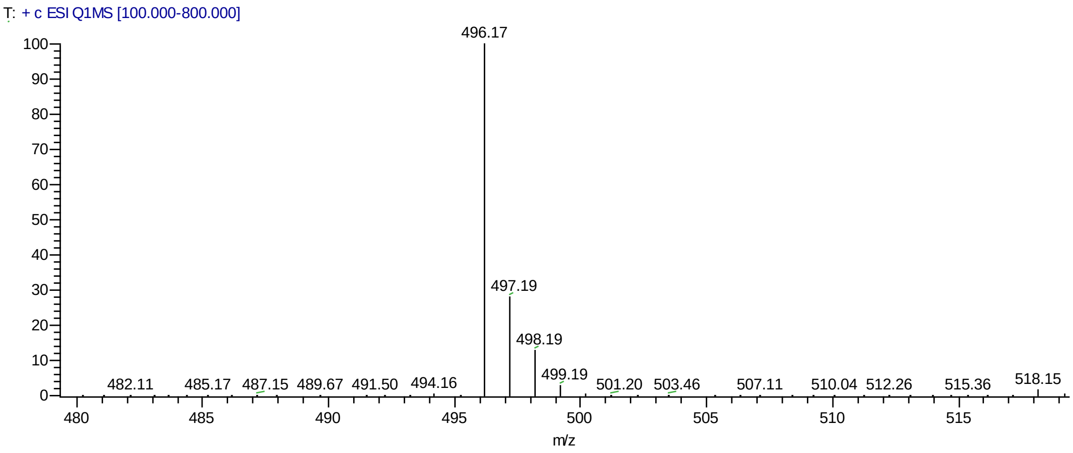

Figure S85. ESI-MS spectrum of the target compound **6q**
